# CYD0281, a Bcl-2 BH4 domain antagonist, inhibits tumor angiogenesis and breast cancer tumor growth

**DOI:** 10.1186/s12885-023-10974-4

**Published:** 2023-05-26

**Authors:** Yihua Lin, Yiling Zhao, Minggui Chen, Zishuo Li, Qiao Liu, Jian Chen, Yi Ding, Chunyong Ding, Ye Ding, Cuiling Qi, Lingyun Zheng, Jiangchao Li, Rongxin Zhang, Jia Zhou, Lijing Wang, Qian-Qian Zhang

**Affiliations:** 1 School of Life Sciences and Biopharmaceutics, Guangdong Pharmaceutical University, Guangzhou, 510006 China; 2 Chemical Biology Program, Department of Pharmacology and Toxicology, University of Texas Medical Branch, Galveston, TX 77555 USA

**Keywords:** CYD0281, B-cell lymphoma 2 (Bcl-2), Bcl-2 homology (BH) domains, BH4 domain antagonist, Apoptosis, Angiogenesis, Breast cancer

## Abstract

**Background:**

B-cell lymphoma 2 (Bcl-2) family proteins are key regulators of apoptosis, which possess four conserved Bcl-2 homologies (BH) domains. Among the BH domains, the BH3 domain is considered as a potent ‘death domain’ while the BH4 domain is required for anti-apoptotic activity. Bcl-2 can be converted to a pro-apoptotic molecule through the removal or mutation of the BH4 domain. Bcl-2 is considered as an inducer of angiogenesis, which can promote tumor vascular network formation and further afford nutrients and oxygen to promote tumor progression. However, whether disrupting the function of the BH4 domain to convert Bcl-2 into a pro-apoptotic molecule could make Bcl-2 possess the potential for anti-angiogenic therapy remains to be defined.

**Methods:**

CYD0281 was designed and synthesized according to the lead structure of BDA-366, and its function on inducing a conformational change of Bcl-2 was further evaluated via immunoprecipitation (IP) and immunofluorescence (IF) assays. Moreover, the function of CYD0281 on apoptosis of endothelial cells was analyzed via cell viability, flow cytometry, and western blotting assays. Additionally, the role of CYD0281 on angiogenesis in vitro was determined via endothelial cell migration and tube formation assays and rat aortic ring assay. Chick embryo chorioallantoic membrane (CAM) and yolk sac membrane (YSM) models, breast cancer cell xenograft tumor on CAM and in mouse models as well as the Matrigel plug angiogenesis assay were used to explore the effects of CYD0281 on angiogenesis in vivo.

**Results:**

We identified a novel potent small-molecule Bcl-2-BH4 domain antagonist, CYD0281, which exhibited significant anti-angiogenic effects both in vitro and in vivo, and further inhibited breast cancer tumor growth. CYD0281 was found to induce conformational changes in Bcl-2 through the exposure of the BH3 domain and convert Bcl-2 from an anti-apoptotic molecule into a cell death inducer, thereby resulting in the apoptosis of vascular endothelial cells.

**Conclusions:**

This study has revealed CYD0281 as a novel Bcl-2-BH4 antagonist that induces conformational changes of Bcl-2 to convert to a pro-apoptotic molecule. Our findings indicate that CYD0281 plays a crucial role in anti-angiogenesis and may be further developed as a potential anti-tumor drug candidate for breast cancer. This work also provides a potential anti-angiogenic strategy for breast cancer treatment.

**Supplementary Information:**

The online version contains supplementary material available at 10.1186/s12885-023-10974-4.

## Introduction

Angiogenesis is a process of formatting new blood vessels from pre-existing ones, which is important for multiple pathological processes [1, 2]. Deregulated angiogenesis affords nutrients and oxygen for cancer growth, and results in important pathological changes in cancer. Currently approved anti-angiogenic drugs, including bevacizumab, sunitinib, pazopanib, endostar, regorafenib, axitinib, sorafenib, ranibizumab, and aflibercept, are primarily targeting vascular endothelial growth factor (VEGF) signaling pathway, and are often used for the treatment of solid tumors in the clinic [3–5]. Tumor angiogenesis is a multiple pathways regulated process [5]. Therefore, the activation of non-VEGF angiogenic pathways may result in resistance to anti-VEGF therapies for cancer patients [6]. The cancer statistics of 2020 in the United States indicate that breast cancer is the most common form of cancer in women and is the second leading cause of death to lung cancer in females [7]. The disordered growth and metastasis of tumor cells are the leading cause of death in breast cancer patients. The development of breast cancer is angiogenesis-dependent, and the capillary network density is considered a prognostic marker for breast cancer [8, 9]. Anti-angiogenic therapy may be a potential therapeutic strategy for breast cancer [10]. However, anti-VEGF signaling pathway therapy alone or in combination with chemotherapy regimens for the treatment of metastatic breast cancer in the clinic has yielded only modest clinical outcomes [11]. In the previous reports, JE Nör, et al. demonstrated that overexpression of B-cell lymphoma-2 (Bcl-2) in microvascular endothelial cells was sufficient to enhance angiogenesis and accelerate tumor growth [12–14]. Therefore, targeting Bcl-2 may provide a potential therapeutic strategy for angiogenesis, and investigation of more effective Bcl-2-based anti-angiogenic agents may offer potential novel breast cancer therapy.

Bcl-2 family proteins are critical regulators of the apoptosis pathways. Based on the presence of Bcl-2 homology (BH) domains (BH1-BH4), the family is structurally subdivided into 3 subtype groups: pro-apoptotic BH3-only subgroup (BH3), pro-apoptotic ‘effectors’ (BH1-BH3) and anti-apoptotic subgroup (BH1-BH4) [15–19]. Bcl-2 is a member of anti-apoptotic molecules that contains four BH domains [20]. The BH1, BH2, and BH3 domains can form a hydrophobic groove, which mediates protein-protein interactions between the Bcl-2 and BH3 domain of pro-apoptotic Bcl-2 family members to form proteins complex, and consequent inhibit the function of pro-apoptotic Bcl-2 family proteins and prevent cell apoptosis [21, 22]. The N-terminal amphiprotic helix BH4 domain (aa 6-31) may be important for a particular conformation of Bcl-2. The BH4 domain does not participate in protein dimerization, but it facilitates the pro-apoptotic regulators to bind to the hydrophobic groove and is necessary for promoting cell survival [19, 23]. Therefore, the BH4 domain is essential for exhibiting the anti-apoptotic function of Bcl-2 [23, 24]. The dysfunction of Bcl-2 is related to several types of diseases, including tumor development [15, 25]. Recently, reports demonstrated that the anti-apoptotic protein Bcl-2 functions as a pro-angiogenic signaling molecule [13, 14]. Therefore, Bcl-2 may be a potential anti-angiogenic therapeutic target for breast cancer therapy. Exploring potential agents to abolish the function of Bcl-2 is essential for anti-angiogenic therapeutic.

Recently, small molecule compounds, which have a structural similarity of BH3 and competition for occupying the hydrophobic groove of Bcl-2 protein with pro-apoptotic BH3 Bcl-2 family members, have been developed to block the anti-apoptosis function of Bcl-2 [26–29]. These BH3-based Bcl-2 inhibitors are a new class of cancer drugs that have been evaluated in pre-clinical and clinical trials [28, 29]. However, ABT-737 and ABT-263, the BH3 mimetic, can induce thrombocytopenia and impair the hemostatic function of platelets [30]. The limited efficacy due to the drug resistance and side effects of BH3 domain antagonists limit the application of cancer therapy in the clinic. Therefore, it is critical to explore new types of small molecule antagonists to target Bcl-2 for tumor therapy.

Reports indicated that the removal or mutation of the BH4 domain can abolish the anti-apoptotic activity of Bcl-2 by converting it from an anti-apoptotic molecule into a pro-apoptotic effector [24, 31–34]. BDA-366, a Bcl-2-BH4 domain antagonist, can induce conformations to change in the exposure of the BH3 domain and abrogate the anti-apoptotic function of Bcl-2, and further inhibit tumor growth of lung cancer and multiple myeloma [35, 36]. Therefore, the BH4 domain became a unique therapeutic target for recent efforts developing anti-cancer therapy through the alteration of Bcl-2 from a survival promoter to a death inducer. Nevertheless, whether the Bcl-2-BH4 domain antagonist can suppress tumor angiogenesis remains unknown. Elucidating the function of the exposure of the Bcl-2-BH3 domain on tumor angiogenesis and exploring new Bcl-2-BH4 domain inhibitors to convert the function of Bcl-2 may provide a potential new strategy for anti-angiogenic therapy of breast cancer.

In this study, we have discovered a small molecule compound CYD0281, a Bcl-2-BH4 domain antagonist, which could significantly inhibit angiogenesis both in vitro and in vivo. Furthermore, CYD0281 could inhibit tumor growth in breast cancer cell xenograft mouse model and experimental tumor model of the chick embryo. Moreover, CYD0281 was found to induce apoptosis of vascular endothelial cells. The effects are likely dependent on the exposure of the BH3 domain of Bcl-2 and abrogating the anti-apoptotic function of Bcl-2, via converting it from an apoptosis inhibitor into a death promoter. The investigations in this study may provide a novel therapeutic strategy by utilizing a new Bcl-2-BH4 domain antagonist to inhibit angiogenesis for breast cancer therapy.

## Materials and methods

### Chemicals and reagents

Bcl-2-BH4 domain antagonist, BDA-366 (S7849), was purchased from Selleck Chemicals (Selleck, Shanghai, China). CYD0281 was designed and synthesized as well as patented (jointly with Emory University) by Dr. Zhou’s laboratory at the University of Texas Medical Branch (UTMB) and was delivered for biological testing under the mutually signed material transfer agreement (MTA) with approval from the Office of Technology Transfer (OTT) of the UTMB. BDA-366 and CYD0281 were dissolved in dimethylsulfoxide (DMSO, Sigma-Aldrich St. Louis, MO, USA). Recombinant Human Bcl-2 Protein (NBP2-34889) was purchased from Novus Biologicals (Littleton, CO, USA). The following primary antibodies were used for immunohistochemical (IHC), immunofluorescence (IF), Western blotting (WB), and immunoprecipitation (IP) assays: rabbit anti-BH3 Domain Specific (AP1303a, diluted at 1:100 for IF and 1:1000 for IP) was purchased from Abgent (San Diego, California, USA), mouse anti-Bcl-2 (15071, diluted at 1:1000 for WB) and rabbit anti-Cytochrome C (4272T, diluted at 1:1000 for WB) were purchased from Cell Signaling Technology (CST, Beverly, MA, USA), rabbit anti-PARP1 (13371-1-AP, diluted at 1:1000 for WB) was purchased from Proteintech, rabbit anti-calnexin (AF2425, diluted at 1:150 for IF) and mouse-anti-Bcl-2 (AG1222, diluted at 1:50 for IF) were purchased from Beyotime (Wuhan, China), rabbit anti-CD31 (ab28364, diluted at 1:100 for IF), rabbit anti-Ki67 (ab15580, diluted at 1:100 for IHC) and rabbit anti-Bcl-2 (ab32124, diluted at 1:50 for co-IP) were purchased from Abcam, and rabbit anti-Mcl1 (PB9132, diluted at 1:1000 for WB), rabbit anti-Bax (AF1270, diluted at 1:1000 for WB), and rabbit anti-Bim (BM4183, diluted at 1:350 for WB) were purchased from Boster (Wuhan, China). Mito-Tracker Red CMXRos (C1035) was purchased from Boster (Wuhan, China). Bcl-2 siRNAs were synthesized by Ribobio Co., Ltd. (Guangzhou, China) and transfected into HUVECs using Lipofectamine RNAiMAX (Invitrogen, Carlsbad, CA, USA) at a final concentration of 100 nM.

### Cell lines

Primary human umbilical vein vascular endothelium cells (HUVECs, ATCC® PCS-100-010™) were purchased from American Type Culture Collection (ATCC, Manassas, USA) and maintained in endothelial cell growth medium including growth supplements (EGM, CC-3124, Lonza, Walkersville, MD, USA). 4T1 mouse breast cancer cell line and MDA-MB-231 human breast cancer cell line were obtained from the cell bank of the Chinese Academy of Sciences (Shanghai, China). The 4T1 cells were maintained in RPIM1640 (Gibco, Carlsbad, CA, USA), and MDA-MB-231 cells were cultured in L-15 (Gibco). Both RPIM1640 and L-15 media contain 10% fetal bovine serum (FBS; Gibco), 100 U/mL penicillin, and 100 μg/mL phytomycin. HUVECs and 4T1 cells were incubated at 37 °C with 5% CO_2_, and MDA-MB-231 cells were incubated at 37 °C without CO_2_.

### Animals and treatment

Sprague-Dawley rats (SD rats, female, 4 ~ 5 weeks old) and BALB/c mice (female, 5 ~ 6 weeks old) were purchased from Guangdong Medical Laboratory Animal Center (Guangzhou, China) and housed in a 12-h light/12-h dark cycle, 24 ± 2 °C, and 50 ~ 60% humidity environmentally condition.

To construct a xenograft tumor model, 4T1 cells (1 × 10^5^ cells/200 μL) were subcutaneously injected into the second right mammary fat pad of the BALB/c mice. After 7 days of tumor cell inoculation, the mice were divided into 3 groups randomly and intraperitoneal injection of DMSO or CYD0281 (10-, 30- and 50-mg/kg body weight) every 2 days for 2 weeks according to the previous report [36]. During tumor growth, the length and width of tumors were measured using calipers, and the tumor volume was calculated as 0.5236 × (length × width^2^). After drug treatment, the mice were sacrificed by cervical dislocation, and the tumors were harvested and weighed.

### Cell viability assay

Cell viability was assessed using a cell counting kit-8 (CCK-8) kit (C0039, Beyotime, Shanghai, China) and BS350B (Biosharp, Hefei, China)). Briefly, HUVECs cells were seeded in 96-well plates at a density of 2 × 10^3^ cells/well (200 μL). Then, the indicated concentrations of BDA-366 and CYD0281 were added to the cells 24 h later. After 48 h incubation, 20 μL CCK8 reagent was added to each well and incubated for 3 h. The optical density was measured at 450 nm using a multimode enzyme plate analyzer (Thermo Fisher Scientific, Inc., Waltham, MA, USA).

### In vitro HUVECs cell migration assay

HUVECs cells were treated with the indicated concentrations of DMSO, BDA-366, and CYD0281 for 48 h. Then, the cells were re-suspended at the concentration of 1.5 × 10^5^ cells/mL in the serum-free EBM-2 medium containing DMSO, BDA-366, or CYD0281, and added to the upper chamber of the Transwell culture system, and then placed to the lower chamber that contained EBM-2 with growth supplements. After 20 h incubation, the cells migrated to the bottom side of the membrane and were fixed with 4% paraformaldehyde for 30 min and stained with 0.1% crystal violet solution for 15 min. Then, cells on the upper surface of the membrane were removed. The membranes were photographed under an inverted microscope and the migrated cells were counted.

### In vitro HUVECs tube formation assay

The pre-treated HUVECs cells were re-suspended in EBM-2 with half-content growth supplements and the indicated concentration of DMSO, BDA-366, or CYD0281, and then added to a Matrigel pre-coated 96-well plate at the density of 3 × 10^4^ cells/well. The cells were further maintained for 5 h at 37 °C with 5% CO_2_ to form a vascular tube. The cells were fixed with 4% paraformaldehyde solution for 30 min at room temperature and the tubes were photographed under an inverted microscope and the length of the tubes was measured.

### In vitro rat aortic ring assay

Aortas were isolated from Sprague-Dawley rats (4 ~ 5 weeks old, female; the Guangdong Medical Laboratory Animal Center, Guangzhou, China) and removal of the outer connective fibro adipose tissue surrounding the aortas and cut into approximately 1 mm thick rings. The rings were randomly put into 48-well plates, which were pre-coated with the growth factor reduced Matrigel, and further sealed with Matrigel. EBM-2 with growth supplements and the indicated concentration of DMSO, BDA-366, or CYD0281 was added to the wells and incubated at 37 °C with 5% CO_2_ for 8 days. The formed microvessel that sprouts from aortic rings was fixed using 4% paraformaldehyde and photographed using an inverted microscope. Microvessel outgrowth was quantified using Image-Pro Plus 6.0 image analysis system (IPP 6.0, Media Cybernetics, Inc., Rockville MD, USA).

### Chick embryo chorioallantoic membrane (CAM) assay

Fertilized eggs of white leghorn chicken were obtained from the Avian Farm of South China Agriculture University (Guangzhou, China). According to the previous report, the eggshells were cleaned with 75% ethanol and incubated under the condition of 60% humidity and 37 ± 1 °C for 7.5 days and randomly divided into DMSO, BDA-366, and CYD0281 groups [37]. Then, a small window (1 cm diameter) was drilled above the air chamber of well-developed eggs with a dental drill, and the shell was peeled off using ophthalmic forceps. The indicated concentrations of compounds (below ~ 0.1% concentration of xenograft tumor model treatment) were prepared by dissolving them in 30 μL of phosphate-buffered saline (PBS, pH 7.4), and were placed directly on CAM of the live chick embryo through the window. After 48 h incubation, the eggshells were sacrificed, and the CAM vasculature around the windows was cut off. The CAMs were inverted and photographed under a stereomicroscope (Olympus SZX16). The percentage of blood vessel area in the total drug-treated area was calculated as microvessel density (MVD) using the IPP 6.0 image analysis system.

### Chick embryo yolk sac membrane (YSM) assay

After 3 days of incubation, the well-developed chick embryos were transferred into sterile dishes, and the vessels were faced upward. Then, the marked silastic rings were placed onto the top of the vessel regions of the yolk sac membrane, and DMSO or indicated concentrations of CYD0281 were added in the center of silastic rings, respectively. The YSM vasculature in the rings was photographed and the MVD was quantified using IPP 6.0 at 0, 12, and 24 h after drug treatment.

### Experimental breast cancer assay

MDA-MB-231 cells were re-suspended at the concentration of 1 × 10^8^ cells/mL, and 50 μL cell suspension was added in the middle of the silastic rings that were placed on the CAM of chick embryos that were prepared as CAM assay indicated above. After 30 h of incubation, the chicken embryos were randomly grouped and treated with DMSO and indicated concentrations of BDA-366 or CYD0281. The drugs were added in the middle of the rings once daily for 3 days. After treatment, the macroscopic tumor could be seen on the CAM in successfully established models and the CAMs were peeled off and photographed. The length and width of the tumor were measured and tumor volume on CAM was calculated as 0.5236 × (length × width^2^). The MVD was calculated using IPP 6.0. Then, the tumors were fixed with 4% polymethylene formaldehyde and embedded with paraffin for hematoxylin and eosin (H&E) staining.

### In vivo Matrigel plug angiogenesis assay

The widely used in vivo Matrigel plug angiogenesis assays for quantifying the angiogenic ability are more easily than chick embryo angiogenesis assays [38]. BALB/c mice (female, 5 ~ 6 weeks old) were purchased from Guangdong Medical Laboratory Animal Center. The mice were randomly divided into two groups and were subcutaneously injected with 0.5 mL of Matrigel (356230, Corning, Bedford, MA, USA) supplemented with 150 ng/mL of fibroblast growth factor-2 (FGF-2, 3139-FB/CF, R&D systems, Minneapolis, MN, USA), 60 Units/mL of heparin (Cisen pharmaceutical company, Shandong, China), and DMSO or CYD0281 (50 μg/mL) according to the previous report [39]. The Matrigel quickly polymerized to form a solid gel and incubated for 7 days in vivo. Then, the mice were killed and the Matrigel plugs were collected and photographed. The angiogenesis in the plugs was analyzed by detecting the expression of CD31, a vascular marker, using an immunofluorescence assay.

### Histological, immunofluorescence, and immunohistochemistry assays

The fixed tumor tissues in CAM and BALB/c mice and Matrigel plugs were embedded in paraffin and cut into 3 μm sections. The sections from tumor tissues from CAM were stained with H&E staining for histological analyses. The sections of tumor tissues in BALB/c mice were incubated with CD34 primary antibody and HRP-conjugated secondary antibody for immunohistochemistry assay to detect tumor angiogenesis. The sections of Matrigel plugs were incubated with CD31 primary antibody to detect angiogenesis in vivo and HUVECs cells were incubated with Bcl-2 (BH3-specific) primary antibody to detect the conformations change in the exposure of the BH3 domain, and then incubated with Alexa Fluor 555-conjected secondary antibody for immunofluorescence assay. DAPI was used to stain the cell nuclei. The MVD was analyzed using the IPP 6.0 image analysis system.

### Western blotting assay

Total proteins from HUVECs that were treated with DMSO, BDA-366, or CYD0281 were harvested using RIPA lysis buffer. Proteins of mitochondrial and cytosolic were extracted using the Cell Mitochondria Isolation Kit (C3601, Wuhan, China) according to the manufacturer’s instructions. All the proteins were examined by 10% sodium dodecyl sulfate-polyacrylamide gel electrophoresis (SDS-PAGE). The separated proteins on the gel were transferred onto polyvinylidene fluoride (PVDF) membranes and incubated with relevant primary antibodies and HRP-conjugated secondary antibodies. Then, protein signals on the membranes were visualized using enhanced chemiluminescence (ECL) chemiluminescent system (#4AW011-200, 4A Biotech, Beijing, China). The intensity of protein bands was quantified using Quantity One software (version 4.6.2 for Windows; Bio-Rad Laboratories, Inc., Hercules, CA, USA). Glyceraldehyde-3-phosphate dehydrogenase (GAPDH) was used as a protein loading control. The signals were visualized using an ECL chemiluminescent system.

### Immunoprecipitation assay

Recombinant Human Bcl-2 Protein was treated with CYD0281 in 1% CHAPS (3-[(3-cholamidopropyl)-dimethylammonio]-1-propanesulfonate) lysis buffer at 4 °C for 2 h, followed by immunoprecipitation (IP) using anti-Bcl2/BH3 domain antibody at 4 °C overnight. Then, protein A/G magnetic beads (25 µL) were added and incubated at room temperature for 1 h. The protein complex was washed 3 times and released from the beads by incubating with eluent (100 μL) for 10 min at room temperature. The eluted proteins were analyzed by western blot using the Bcl-2 antibody indicated above.

### Apoptosis analysis by flow cytometry

HUVECs were treated with DMSO, BDA-366, or CYD0281 and then collected after 24 h. The apoptosis cells were detected using the Annexin V-FITC Apoptosis Detection kit (#C1062M, Beyotime Shanghai, China) according to the manufacturer’s introduction. The collected cells were gently resuspended by adding Annexin V-FITC (5 μL), propidium iodide (10 μL), and Annexin V-FITC binding solution (195 μL) for 20 min at room temperature in the dark, and then assessed by fluorescence-activated cell sorter (FACS) analysis on a FACS Calibur flow cytometer (BD Biosciences, San Jose, CA, USA). The percentage of apoptotic cells was analyzed using FlowJo software (FlowJo LLC; Ashland, OR, USA). All data are represented as the mean ± standard deviation (SD).

### Statistical analyses

All data are represented as the mean ± standard deviation (SD). The statistical significance of differences between the two groups was determined with a two-sided Student’s *t*-test at *P* < 0.05 and analyzed using GraphPad Prism 5 (San Diego, CA, USA).

## Results

### CYD0281 is a new potent Bcl-2 BH4 domain antagonist

**Fig. 1:**
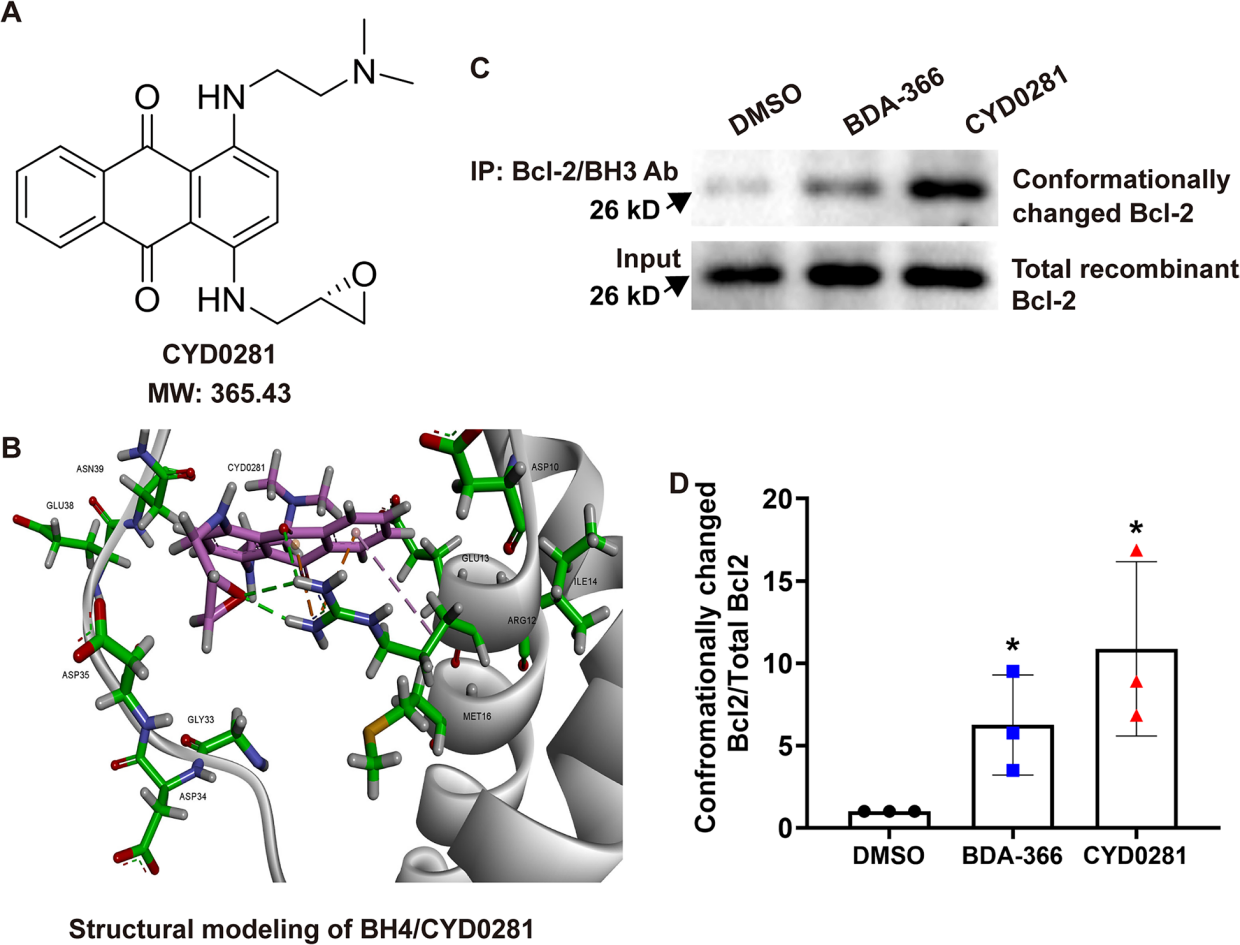
CYD0281 binds to the Bcl-2 BH4 domain to induce the exposure of the BH3 domain. **A** The chemical structure of CYD0281. **B** Structural modeling of CYD0281 docked in the BH4 domain binding pocket of Bcl-2 protein. **C** The exposure of the BH3 domain of recombinant Bcl-2 protein was analyzed by immunoprecipitation followed by Western blot analysis. DMSO and BDA-366 were used as negative and positive control respectively. **D** Quantification of BH3 domain exposed Bcl2, *n* = 3. Significant effect compared to the DMSO group: *P < 0.05

To explore a new compound by targeting the Bcl-2 BH4 domain, we have designed and synthesized CYD0281 according to the structure of BDA-366, a Bcl-2 BH4 domain antagonist, through structure-based and computer-aided drug design (Fig. 1A). Then, the structure modeling of BH4 domain (PDB ID codes: 1G5M and 1G5O) and CYD0281 was analyzed using the University of California, San Francisco (UCSF) DOCK 6.1 program suite as previously described [36, 40] (Fig. 1B). BDA-366 was reported to negatively regulate Bcl-2 activity through binding with the BH4 domain and further induce a conformational change of Bcl2. To explore whether CYD0281 may result in a similar conformational change in Bcl-2, an in vitro cell-free assay was employed. Purified recombinant Bcl-2 protein was treated with CYD0281 (0.5 μM) or BDA-366 (positive control, 0.5 μM) in 1% CHAPS lysis buffer. Then, an anti-Bcl2/BH3 domain antibody was used in IP, and the Bcl-2 antibody was used in western blotting to detect a conformational change in the BH3 domain of Bcl-2 as previously reported [36]. Results indicated that CYD0281 enhances the ability of Bcl2/BH3 domain-specific antibody bind to Bcl-2 protein as BDA-366 (Fig. 1C). In addition, CYD0281 leads to greater exposure of the Bcl-2 BH3 domain than BDA-366 at the same drug concentration (Fig. 1D). These findings provided direct evidence that CYD0281 can bind with the BH4 domain to induce a conformational change of Bcl-2 and result in greater exposure of the BH3 domain compared with BDA-366.

### CYD0281 inhibits cell viability and promotes apoptosis of vascular endothelial cells by inducing a conformational change of Bcl-2

**Fig. 2:**
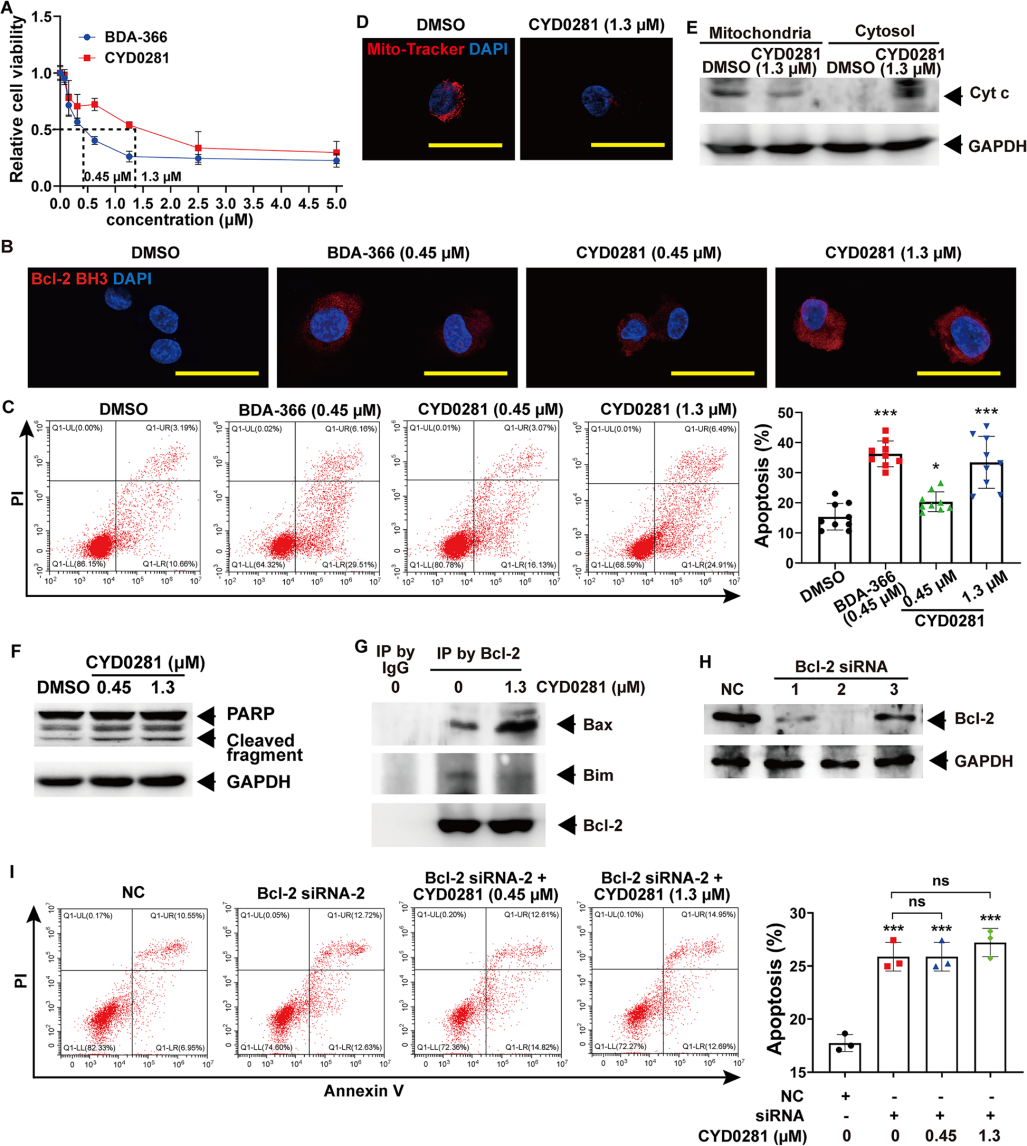
CYD0281 promotes HUVECs cell apoptosis by the exposure of the Bcl-2 BH3 domain. **A** Cell viability of HUVECs at 48 h. The exposure of the BH3 domain of Bcl-2 detected by immunofluorescence (IF) staining using anti-Bcl-2/BH3 domain antibody (**B**), and flow cytometry analysis of cell apoptosis (**C**) in HUVECs treated with BDA-366 and CYD0281 at the IC_50_ concentration. HUVECs were treated with CYD0281 at the IC_50_ concentration for 48 h, mitochondrial dysfunction was analyzed by Mito-Tracker Red CMXRos staining (**D**), Cyt c release (**E**) and PARP cleavage (**F**) were analyzed by western blotting assay, and Bcl-2 associated Bax or Bim was analyzed by co-IP using Bcl-2 antibody (**G**). **H** The expression of Bcl-2 in HUVECs was inhibited using siRNAs (1, 2, and 3) and analyzed by western blotting assay. **I** Flow cytometry analysis of cell apoptosis in Bcl-2-silenced HUVECs that were treated with CYD0281 (*n* = 3). Significant effect compared to the DMSO group: *P < 0.05 and ***P < 0.001. ns: no significant differences. Scale bars: 50 μm

Based on the exposure of the Bcl-2 BH3 domain that can lead to cell apoptosis, we first investigated whether antagonizing the Bcl-2 BH4 domain can inhibit the viability of HUVECs, focusing on a drug concentration range from 0.01 μM to 10 μM (DMSO concentration was kept below 0.1%). Both CYD0281 and BDA-366 significantly inhibit cell viability of HUVECs in a concentration-dependent manner with 50% inhibition at 1.3 μM of CYD0281 and 0.45 μM of BDA-366 (Fig. 2A). In addition, immunofluorescence was used to further confirm whether Bcl-2 BH4 domain antagonist induced Bcl-2 conformational change in HUVECs using Bcl-2 BH3 domain-specific antibody according to the previous report [36]. As shown in Fig. 2B, the fluorescence signaling was undetectable in DMSO-treated HUVECs cells and significantly enhanced in CYD0281- and BDA-366-treated cells. In addition, the fluorescence intensity in CYD0281-treated cells was in a drug concentration-dependent manner. Meanwhile, the fluorescence signaling in CYD0281-treated cells was higher than that in BDA-366-treated cells, when HUVECs cells were treated with the drug at the same concentration. Then, HUVECs cells were treated with CYD0281 (0.1 μM, 0.45 μM, and 1.3 μM) or BDA-366 (0.1 μM and 0.45 μM), and cell apoptosis was further determined by flow cytometry combined with Annexin V-FITC/propidium iodide (PI) staining and western blotting assay. The results indicated that cell apoptosis was increased in cells treated with CYD0281 or BDA-366 compared with that of the DMSO group (Fig. 2C and Supplementary Fig. 1A). The statistical analysis showed that the apoptosis rate of CYD0281- or BDA-366-treated cells was significantly increased than that of DMSO-treated cells, and the increase of apoptosis rate induced by CYD0281 was in a concentration-dependent manner in HUVECs (Fig. 2C). These findings provided direct evidence that CYD0281 induces exposure of the BH3 domain of Bcl-2 and results in cell apoptosis.

Our results showed that Bcl-2 is mainly expressed in the cytoplasm and mitochondria using immunofluorescence staining (Supplementary Fig. 1B). Meanwhile, CYD0281 reduced Mito-Tracker staining and promote the release of cytochrome c (Cyt c) from mitochondria to the cytosol in HUVECs (Fig. 2D and 2E). Furthermore, CYD0281 significantly increased the PARP cleavage in HUVECs (Fig. 2F). All the results showed CYD0281 resulted in mitochondrial dysfunction and apoptosis in HUVECs like the BDA-366 effect on lung cancer cells in our previous report [36]. Our previous report indicated that BH3-exposed Bcl-2 possesses a greater ability to interact with Bax than Bim and further induced activation of Bax’s cell-killing function [36]. Therefore, co-IP assay was used to further investigate whether the interaction between Bcl-2 and Bax or Bim was affected by CYD0281 in HUVECs. The results revealed that CYD0281 enhanced the interaction between Bcl-2 and Bax in association with the inhibited binding of Bcl-2 with Bim (Fig. 2G). In addition, CYD0281 and BDA-366 were found not to significantly regulate the expression of Bcl-2, Bax, Bim, and Mcl-1 in HUVECs (Supplementary Fig. 1C and D). Nevertheless, treatment with DBA-366, but not CYD0281, significantly inhibited the expression of Bcl-2 in HUVECs cells (Supplementary Fig. 1C and D). The results above suggest that CYD0281 increased cell apoptosis may be dependent on the induction of Bcl-2 conformational change to expose the BH3 domain, and further promote the Bcl-2/Bax interaction to activate Bax.

Additionally, inhibition of Bcl-2 using siRNA significantly induces cell apoptosis in HUVECs, and further treatment with CYD0281 in Bcl-2-silenced HUVECs cannot further increase cell apoptosis (Fig. 2H and I). The above findings suggest that the Bcl-2 BH4 domain antagonist-induced apoptosis is likely caused by the exposure of the Bcl-2 BH3 domain and induced Bcl-2-dependent Bax activation. These findings suggested that CYD0281 negatively regulates cell survival by inducing a conformational change of Bcl-2 but not suppressing its expression in vascular endothelial cells.

### CYD0281 suppresses microvessel formation in vitro

**Fig. 3:**
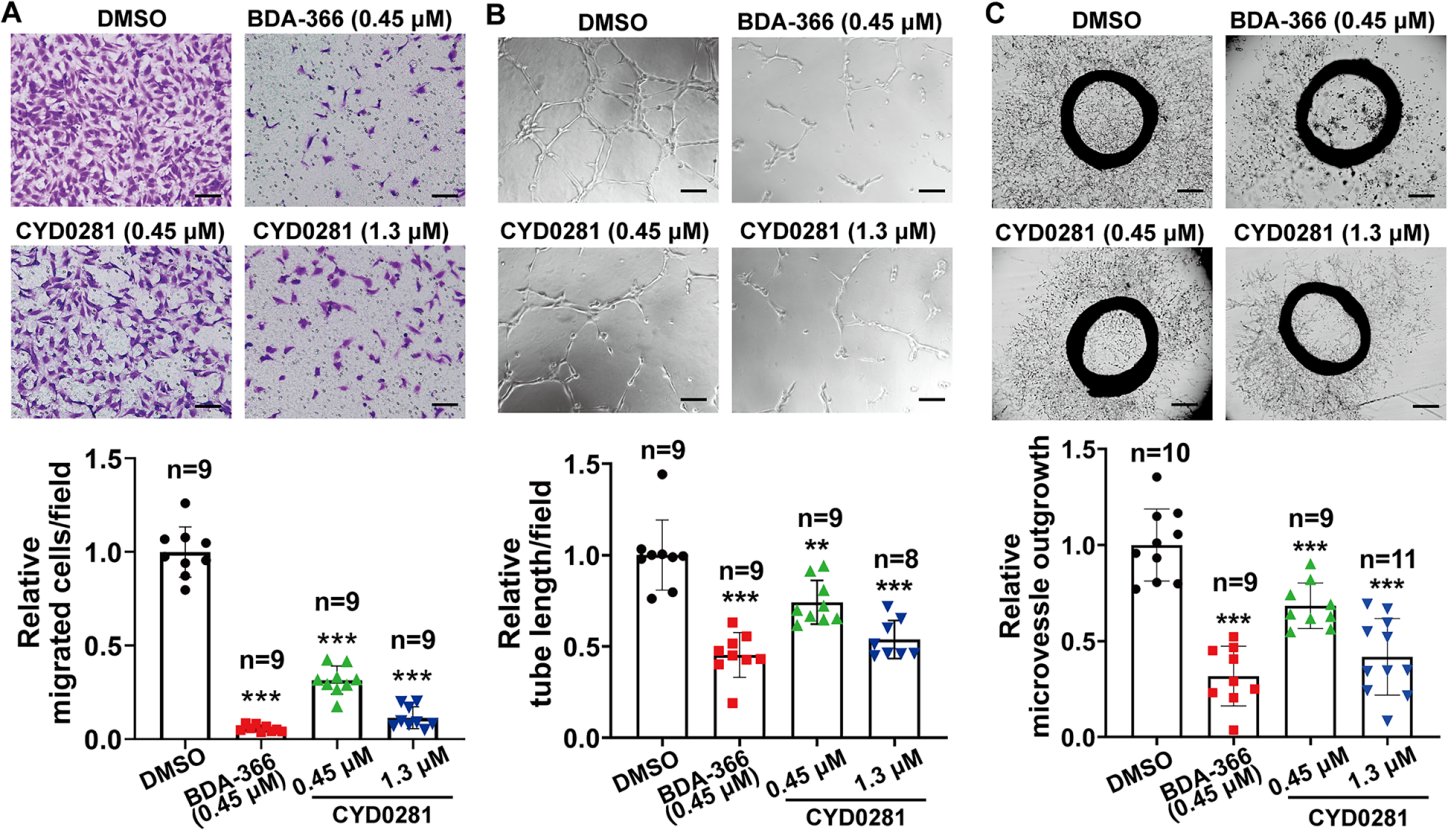
CYD0281 inhibits angiogenic differentiation of HUVECs in vitro. Representative microscopic images and quantification of cell migration (**A**), and tube formation in Matrigel (**B**). **C** Representative images of endothelial cells sprout and quantification from rat aortic ring assay at 8 days of treatment. Significant effect compared to the DMSO group: **P < 0.01 and ***P < 0.001. Scale bars: 100 μm (**A** and **B**) and 500 μm (**C**)

Given that Bcl-2 functions as a pro-angiogenic molecule and regulation of vascular endothelial cell apoptosis are critical for angiogenesis [41], cell migration assay and tube formation assay were used to examine the function of CYD0281 on the regulation of angiogenesis. As shown in Fig. 3A and B, both CYD0281 and BDA-366 markedly inhibited cell migration and the formation of capillary-like microtubule networks in HUVECs compared to those treated with DMSO. In addition, CYD0281 and BDA-366 at 0.1 μM concentration were observed to inhibit cell migration of HUVECs compared to the DMSO group (Supplementary Fig. 1D). These results indicate that the anti-angiogenic effect may be dependent on the CYD0281-induced pro-apoptosis function.

Then, the microvessel formation from in vitro rat aortic rings was detected to further confirm the directly anti-angiogenic role of CYD0281 and BDA-366. The microvessels were formed by outgrowth epithelial cells from 1- to 1.5-mm long segments of rat aorta that put in Matrigel was significantly suppressed by CYD0281 and BDA-366 (Fig. 3C). All the results showed the anti-angiogenic effect of CYD0281 in both HUVECs and rat aorta rings was in a concentration-dependent manner. However, the inhibitory effect of BDA-366 was better than CYD0281 at the concentration of 0.45 μM (IC_50_ of BDA-366). Together, these results demonstrated that the Bcl-2 BH4 domain is a potential target for anti-angiogenic therapeutics.

### CYD0281 suppresses the angiogenesis in chick embryo CAM and YSM models

The vascularization in chick embryos CAM and YSM models are becoming the most widely used models for studying the formation of blood vessels in vivo. We therefore further explored CAM and YSM models to confirm whether CYD0281 possesses anti-angiogenic activity in vivo. DMSO, BDA-366, or CYD0281 (1 μg/egg, which is the dose of 0.1% that was used in vivo experiment of BDA-366 according to the previous report [36]) was added to the chick embryo CAM and YSM to determine the effect of Bcl-2 BH4 domain on angiogenesis. Both BDA-366 and CYD0281 significantly resulted in the reduction of blood vessel branches in CAM and YSM models compared to the DMSO group (Supplementary Fig. 2).

**Fig. 4:**
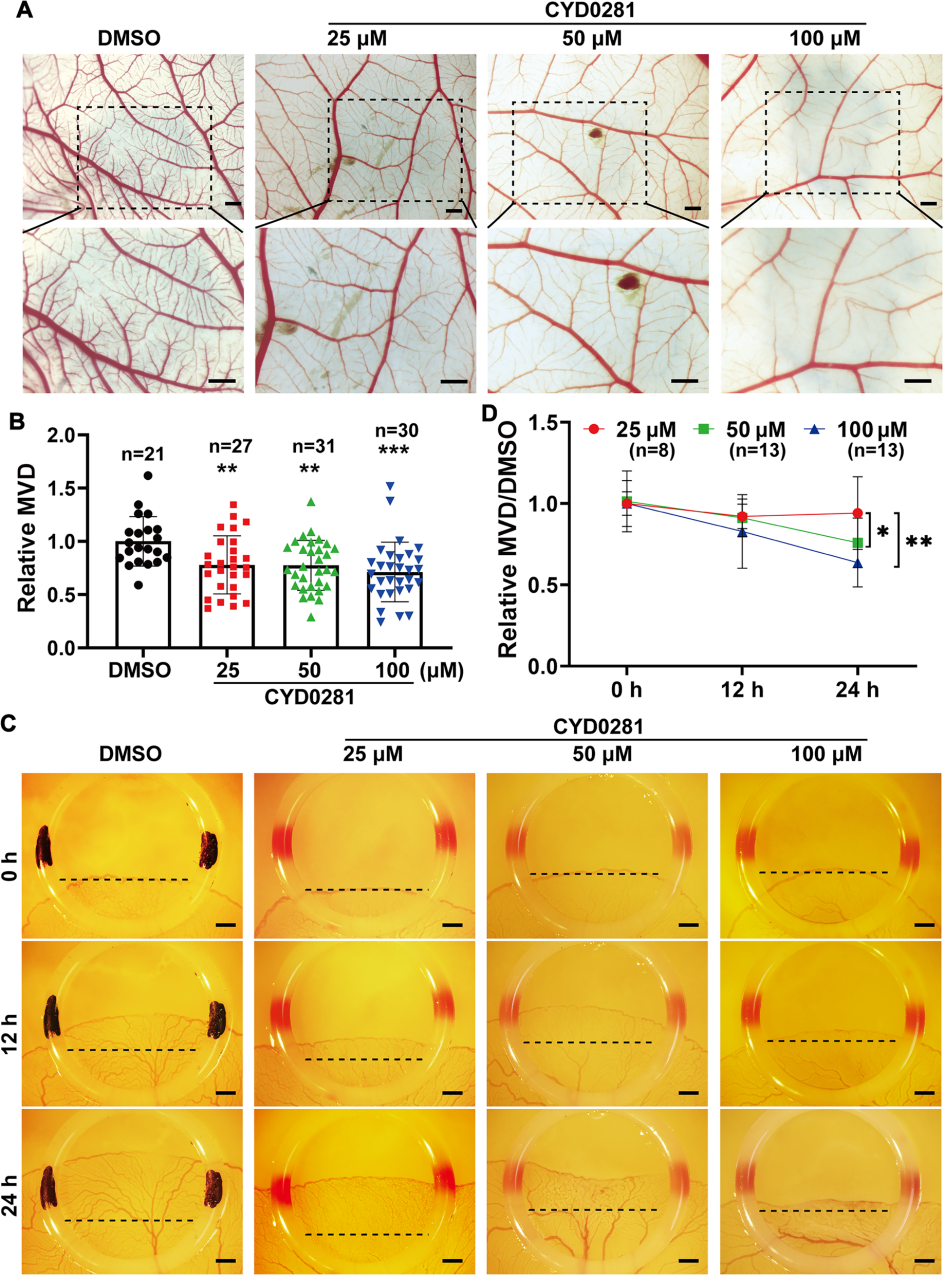
CYD0281 inhibits angiogenesis in a concentration-dependent manner in chick embryo chorioallantoic membrane (CAM) and yolk sac membrane (YSM) models. **A** Representative images of the growth of blood vessel branches and vascular network in chick embryo CAM model at 48 h of incubation. **B** Quantification of microvessel density (MVD). **C** Angiogenesis assay in chick embryo YSM model at 24 h of incubation. **D** Quantification of relative MVD/DMSO group in YSM models. Significant effect compared to DMSO group (**B**) and between the groups (**D**): *P < 0.05, **P < 0.01, and ***P < 0.001. Scale bars: 500 μm (**A**) and 200 μm (**C**)

Furthermore, DMSO or different concentrations of CYD0281 (25, 50, and 100 μM) were added to the chick embryo CAM and incubated for 48 h to determine the inhibitory effects of CYD0281 on angiogenesis. Abundant tiny capillaries network can be seen in the DMSO group and 25 μM CYD0281 group; however, the reduction of blood vessel branches was notably observed at the site of drug administration in the groups treated with CYD0281 at concentrations of 50 μM and 100 μM (Fig. 4A). In addition, the density of the microvessel plexus in the CAM of CYD0281 groups at concentrations of 50 μM and 100 μM were significantly lower than the group treated with DMSO (Fig. 4B).

In addition, different concentrations of CYD0281 as in CAM were exposed to chick embryo YSM to further confirm its anti-angiogenic capability. A density honeycomb network consistency of capillary plexus in the YSM was observed in the DMSO group and 25 μM CYD0281 group; however, the growth of blood vessel network and tiny capillaries in the YSM of CYD0281 groups at concentrations of 50 μM and 100 μM were significantly lower than DMSO group (Fig. 4C). The statistical analysis showed that the blood vessel density and the distance of blood vessel growth in the groups of CYD0281 at concentrations of 50 μM and 100 μM were significantly reduced compared with that group treated by DMSO (Fig. 4D). Moreover, the anti-angiogenic efficacy of CYD0281 in the chick embryo CAM and YSM was also in a concentration-dependent manner. These data further confirmed that inhibiting the Bcl-2 BH4 domain by CYD0281 and BDA366 can suppress angiogenesis in vivo.

### CYD0281 decreases in vivo Matrigel plug angiogenesis

**Fig. 5:**
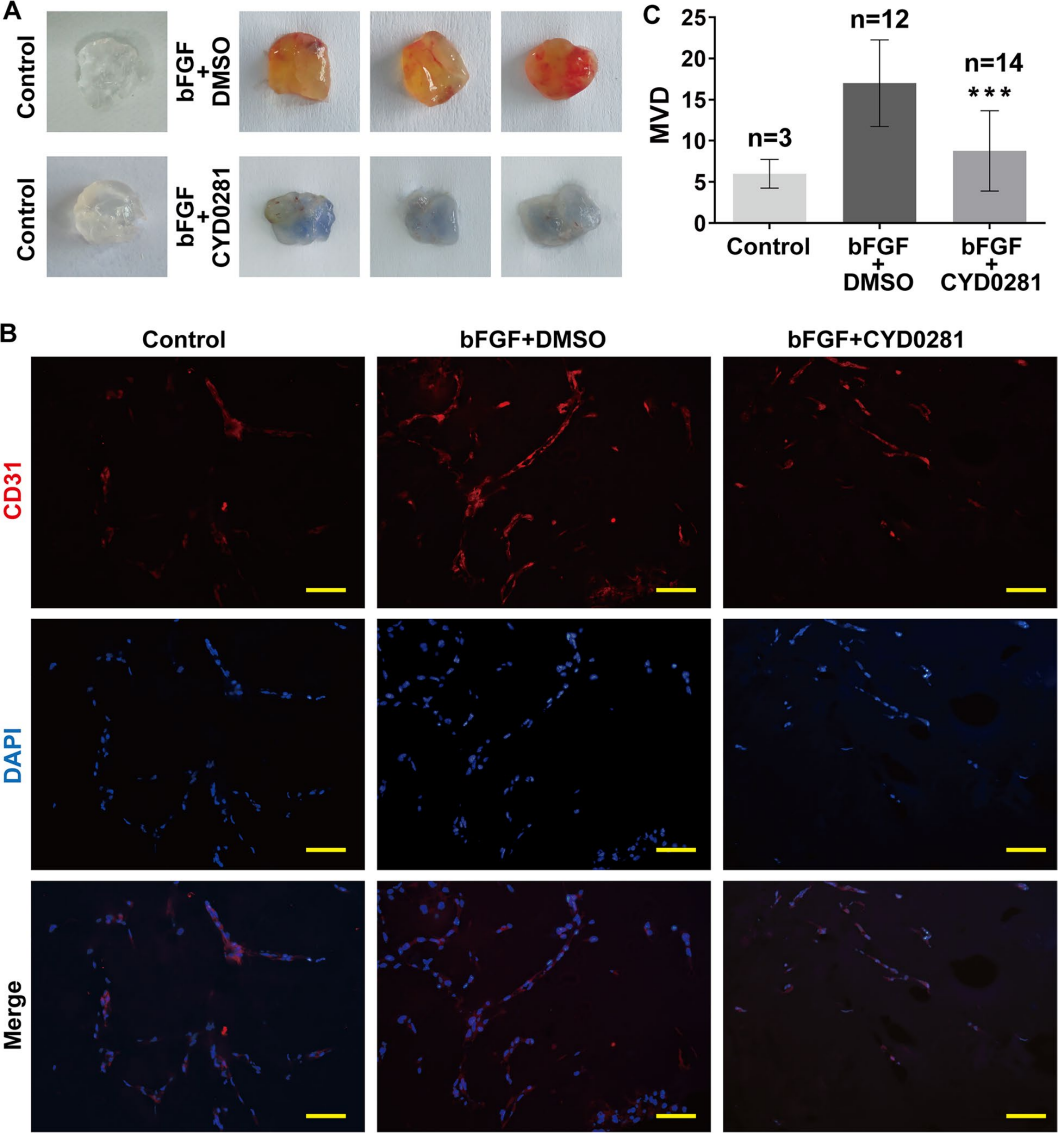
CYD0281 inhibits neovascularization in mouse subcutaneous Matrigel plug model. **A** Representative macroscopic images of Matrigel plugs. **B** Representative immunofluorescence (CD31) images of Matrigel plug tissue sections. **C** Quantification of microvascular density in Matrigel plugs analyzed from image **B**. Significant effect compared to DMSO group: ***P < 0.001; Scale bars: 50 μm

Matrigel plug angiogenesis assay is a technique for quantitative assessment of newly formed blood vessels in the gel plugs from pre-existing vascular in the tissue microenvironment in vivo [42]. Therefore, Matrigels mixed with FGF-2 in combination with CYD0281 or DMSO was subcutaneously injected into Balb/c mice to further detect the effect of CYD0281 on neovascularization in vivo. After 1 week of incubation, gel plugs were harvested and vasculature was examined by staining for CD31 and detected the concentration of hemoglobin in Matrigel. As shown in Fig. 5A, the gel plugs with DMSO were dark-red in color; however, a little light-red in color can be seen in the gel plugs with CYD0281. The results indicated that CYD0281 can inhibit vascular endothelial cells in tissue microenvironment growth into gel plugs. Therefore, we detected the expression of CD31, a marker of vascular endothelial cells, in sections from the harvested gel plugs by immunofluorescence staining assay. It was observed that CYD0281 significantly inhibits the number of CD31-positive cells (red) in gel plugs compared with the DMSO group (Fig. 5B). Furthermore, the MVD in sections of gel plugs with CYD0281 was significantly lower than that in the DMSO group by statistical analysis (Fig. 5C). All the results further confirmed that CYD0281 can inhibit angiogenesis.

### CYD0281 impedes angiogenesis and tumor growth of experimental breast cancer in a chick embryo CAM model

**Fig. 6:**
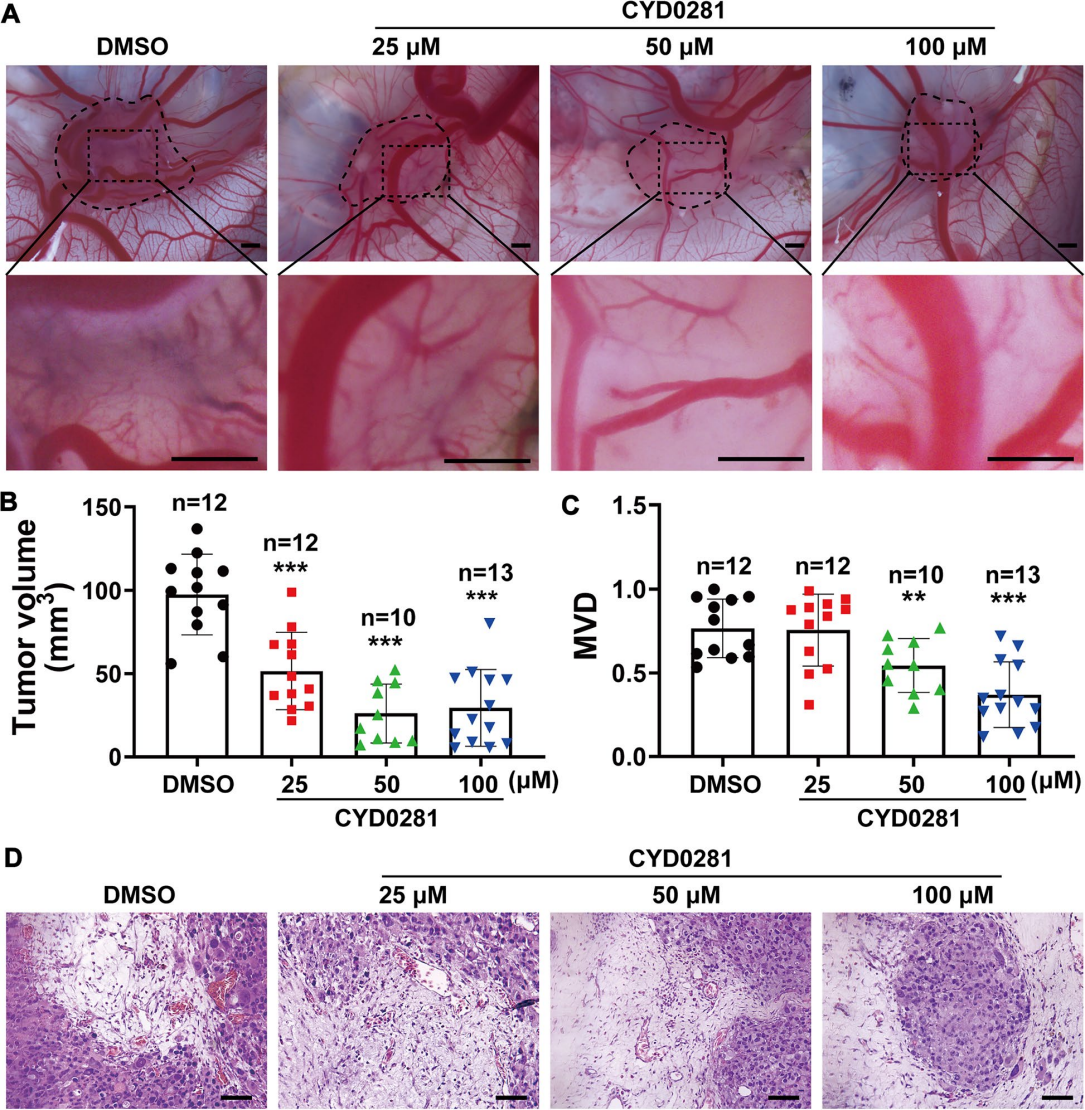
CYD0281 impedes tumor growth and angiogenesis in the chick embryo chorioallantoic membrane (CAM) model in a concentration-dependent manner. **A** Representative images of breast cancer xenografts in CAM assay. Quantification of tumor volume (**B**) and microvessel density (MVD) (**C**) in tumor surface area. **D** Representative H&E stained tumor tissue sections of chick embryo CAM assay. Significant effect compared to the DMSO group: **P < 0.01 and ***P < 0.001. Scale bars: 1600 μm (**A**) and 50 μm (**D**)

Neo-vascularization is essential for tumor development and controlling angiogenesis is a promising method for cancer therapy. Experimental tumor in a chick embryo CAM model is a well-established system to study angiogenesis-dependent tumor growth in vivo [43]. MDA-MB-231 human breast cancer cells were seeded on chicken embryo CAM and treated with DMSO, BDA-366, or CYD0281 at the indicated concentration for 3 days. The results showed that tumor volume and blood vessels in the surface of tumors of the DMSO group were larger than that in BDA-366- and CYD0281-treated groups (Supplementary Fig. 3A and B), and the inhibitory effect of CYD281 was in a concentration-dependent manner (Fig. 6A). In addition, the statistical analyses revealed that tumor volume, which was treated with BDA-366 and CYD0281 was significantly decreased compared with those treated with DMSO (Supplementary Fig. 3C and Fig. 6B). In addition, the MVD was markedly suppressed by BDA-366 and CYD0281 and the inhibitory effect of neovascularization was also concentration-dependent (Supplementary Fig. 3D and Fig. 6C). Then, the tumor tissues were peeled off and embedded for H&E staining. Multiple neovascularizations can be seen in the tumor tissues of the DMSO group, and BDA-366 and CYD0281 significantly attenuated vascular vessels in tumor tissues (Supplementary Fig. 3E and Fig. 6D). All the findings demonstrated that BDA-366 and CYD0281 inhibit tumor growth and angiogenesis during breast cancer development.

### CYD0281 inhibits tumor growth of breast cancer subcutaneous implant tumor model

**Fig. 7:**
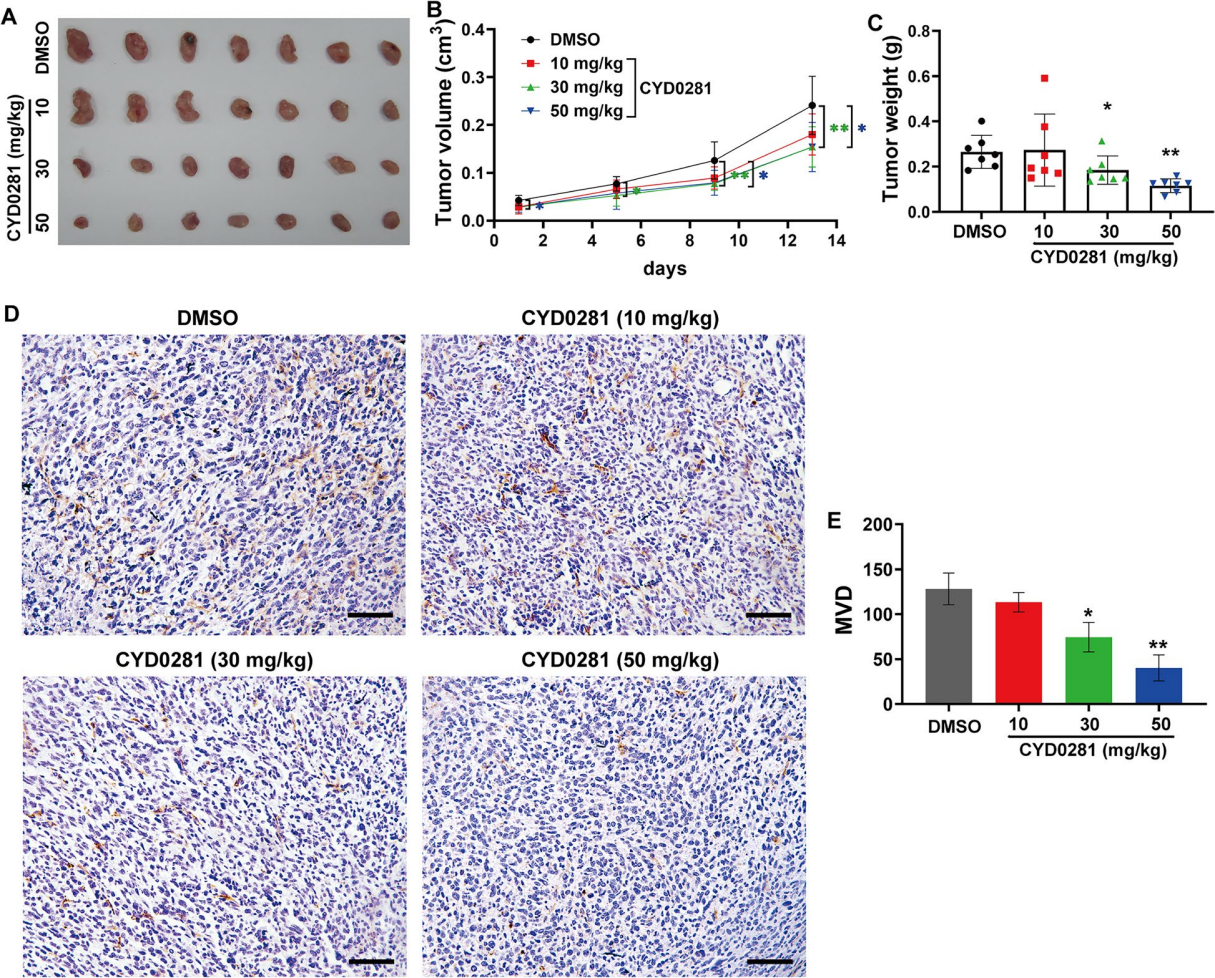
CYD0281 suppresses tumor growth and angiogenesis of 4T1 breast cancer cells subcutaneous implant tumor model. **A** Representative macroscopic images of tumors. Quantification of tumor volume (**B**), and tumor weight (**C**). **D** CD31 immunohistochemistry images of tumor tissue sections. **E** Quantification of microvessel density (MVD). Significant effect between the groups (**B**), and compared to DMSO group (**C** and **E**): *P < 0.05 and **P < 0.01. Scale bars: 100 μm

Based on the inhibition of tumor growth and angiogenesis in the chick embryo CAM model, the 4T1 breast cancer cell subcutaneous xenograft tumor model was employed to further elucidate the possible anti-tumor role of CYD0281 in vivo. The 4T1 cell subcutaneous tumor model mice were constructed and randomly divided into 3 groups, and further treated with DMSO or CYD0281 at 10-, 30-, or 50-mg/kg body weight. During tumor growth, the tumor volumes were measured and CYD0281 at the dose of 30- and 50-mg/kg body weight significantly inhibit tumor growth compared with those in the DMSO group (Fig. 7A and B). After treatment, the tumor tissues were peeled off and weighed, and CYD0281 (30- and 50-mg/kg body weight) markedly inhibited tumor weight compared with the DMSO group (Fig. 7C). Then, the normal tissues and tumor tissues were fixed and embedded for further studies. The neovascularization in tumor tissues was detected through CD31 staining. The number of CD31-positive cells was notably decreased by CYD0281 in the treatment dose of 30- and 50-mg/kg body weight compared with those in the DMSO group (Fig. 7D and E). The effect of NY0123 on breast cancer cells was further detected and showed that CYD0281 significantly inhibits cell viability of MDA-MB-231 cells (Supplementary Fig. 4A), and induces cell apoptosis, PARP cleavage, and Bcl-2 BH3 domain exposure in MDA-MB-231 cells (Supplementary Fig. 4B-D). Importantly, the expression of Bcl-2 in normal tissues was detected using an IHC assay. It was shown that no significant expression of Bcl-2 could be observed in normal tissues (Supplementary Fig. 5A). In addition, the histopathology assay using H&E staining of harvested normal tissues (heart, liver, lung, brain, spleen, kidney, and intestine) showed that no treatment-associated normal tissue toxicities can be seen in the group of CYD0281 at the dose of 30- and 50- mg/kg body weight every 2 days (Supplementary Fig. 5B). Together, these data suggest that CYD0281 exhibits a strong inhibitory effect on tumor angiogenesis and growth of breast cancer with no observed serious toxicity.

## Discussion

Breast cancer is a vascularity-dependent tumor and the increased angiogenesis is linked to an independent adverse prognostic factor in early breast cancer [44]. Therefore, the anti-angiogenic approach is an effective target for breast cancer therapy [45]. Recent clinical trials indicate that current anti-angiogenic treatment targeting VEGF/VEGFR alone or in combination with chemotherapy cannot provide a survival advantage for breast cancer patients, which may be attributed to the feedback activation of non-VEGF angiogenic pathways [6, 11, 46]. Although alone or combination therapy with chemotherapy and target therapy improves the outcome of patients with breast cancer, survival of patients with metastasis remains poor [47]. Angiogenesis is essential for breast cancer metastasis and recurrence [48]. Hence, there is an urgent need to explore more effective anti-angiogenic agents with novel action modes for breast cancer therapy. Previous reports suggest that Bcl-2 expressed in microvascular endothelial cells possesses the pro-angiogenic role [12–14]. We further demonstrated that Bcl-2 BH4 inhibitors, BDA-366 and CYD0281, significantly reduced the angiogenesis of microvascular endothelial cells in this work. In addition, inhibiting Bcl-2 via its BH4 domain using CYD0281 can decrease angiogenesis and breast cancer tumor volume. Based on our findings, we propose that the Bcl-2 BH4 domain may be a target for the therapy of breast cancer. Recently report indicated that combination therapies for breast cancer have the potential to enhance the response to existing drugs [49]. BH3 mimetics in combination with taxanes were efficient for breast cancer patients with paclitaxel-resistant and presented the potential effectiveness of combination targeting Bcl-2 with chemotherapy [50]. Therefore, we speculate that CYD0281 alone or in combination with existing clinical therapeutic regimens may offer an effective strategy to improve the prognosis for breast cancer treatment.

The anti-apoptosis Bcl-2 family members possess Bcl-2 homology domains (BH, BH1 ~ BH4). Structural analyses indicated that the BH1, BH2, and BH3 domains are crucial for proapoptotic activity, and the BH4 domain is essential for anti-apoptotic activity [51]. Bcl-2 plays the anti-apoptotic function by two distinct mechanisms [52, 53]. On the one hand, at the mitochondria, the BH1 ~ 3 domains of Bcl-2 form a hydrophobic pocket that can bind pro-apoptotic proteins and prevent their oligomerization. The BH3 mimetic agents, such as ABT-263 and ABT-199, competitively bind to the pocket and thereby displacing pro-apoptotic proteins to function as anti-Bcl-2 agents [22, 54]. On the other hand, at the endoplasmic reticulum (ER), the BH4 domain of Bcl-2 mediates interaction with the inositol 1,4,5-trisphosphate (IP3) receptor (IP3R) to inhibit IP3-mediated pro-apoptotic Ca^2+^ signals [55, 56]. In addition, phosphatase calcineurin, Raf-1 kinase, and CED-4 have also been reported as potential candidates that bind to BH4 and thereby prevent apoptosis [57–60]. Our previous report indicated BDA-366 (a small-molecule Bcl2-BH4 domain antagonist) could bind to the BH4 domain and induce conformational changes in the exposure of the BH3 domain and abrogate the anti-apoptotic function of Bcl-2 through enhancing the Bcl-2/Bax interaction and further result in inducing Bcl-2-dependent Bax activation and mitochondrial dysfunction, and inhibiting Bcl-2/IP3R interaction and further inducing Ca^2+^ release in lung cancer cells [36]. CYD0281 is a close analog of BDA-366 with the same chemical structural scaffold by a slight modification of one amino side chain of compound BDA-366 by removal of the chiral hydroxy group from the side chain. Such a minor structure modification simplified the chirality of BDA-366 but did not change the key residue interactions for the BH4 binding based on molecular docking. In this work, CYD0281 showed a significantly enhanced effect on upregulating the conformationally changed Bcl-2 level in comparison with BDA-366. Like BDA-366, CYD0281 also induced apoptosis of microvascular endothelial cells in this work. We found Bcl-2 is mainly expressed in the cytoplasm and mitochondria of HUVECs. Meanwhile, this study demonstrated that CYD0281 can induce the exposure of the BH3 domain of Bcl-2 and further result in the increase of Bcl-2 interaction with Bax and mitochondrial dysfunction in HUVECs.

BH3 mimetics and BH4 antagonists have been designed based on the structure of Bcl-2 and studied in pre-clinical and clinical trials [22, 28, 36]. Both BH3 mimetics and Bcl-2 BH4 antagonists can disrupt the antiapoptotic function of Bcl-2 through different mechanisms. The BH3 mimetic agents mimic the BH3-only proteins to play the pro-apoptotic function through the competitive combination to the hydrophobic groove of Bcl-2 with BH3 Bcl-2 family members and then released these pro-death proteins to induce apoptosis [61, 62]. Bcl-2 BH4-selective antagonists are designed to target the BH4 domain and further expose the BH3 domain and convert Bcl-2 into a pro-apoptotic protein, which is functionally different from BH3 mimetics [35, 36]. However, reports indicated that BH3 mimetic drugs (e.g., ABT-737 and ABT-263) may cause adverse effects such as severe thrombocytopenia, neutropenia, and calcium-signaling dysregulation [63–65]. BDA-366, a Bcl-2 BH4-selective antagonist, was found to induce conformational changes in the exposure of the BH3 domain and abrogate the anti-apoptotic function of Bcl-2, and further significantly anti-tumor activity without platelet reduction in lung cancer and diffuse large B-cell lymphoma [35, 36]. Importantly, overexpression of Bcl-2 in microvascular endothelial cells was sufficient to enhance angiogenesis and accelerate tumor growth [12–14]. Collectively, Bcl-2 BH4 domain selective antagonists may provide a potential therapeutic strategy for angiogenesis and serve as novel anti-tumor therapeutic drugs.

In this work, we report that CYD0281, a close analog of BDA-366, acts as a new inhibitor of the Bcl-2 BH4 domain discovered through the computer-assisted structure-based drug design. CYD0281 was found to show a significantly enhanced effect on upregulating the conformationally changed Bcl-2 level in comparison with BDA-366 in HUVECs cells. The previous report indicated that BDA-366 induces cell-death properties and also possesses other mechanisms beyond switching Bcl-2 conformation through downregulating Mcl-1 and/or dephosphorylation of Bcl-2 [66]. However, BDA-366 and CYD0281 were found not to regulate the expression of Mcl-1 in HUVECs. Therefore, CYD0281 as a new Bcl-2 BH4 antagonist promoted cell apoptosis through the induced conformational changes of Bcl-2 in HUVECs and breast cancer cells. Our findings demonstrated that CYD0281 is a potential Bcl-2 BH4 antagonist as a useful research tool inhibitor and a promising therapeutic agent for angiogenesis and breast cancer.

Previous reports indicated that BDA-366 induces cell death through switching Bcl-2 conformation or downregulating Mcl-1 and/or dephosphorylation of Bcl-2 [36, 66]. Herein, we demonstrated that CYD0281 and BDA-366 induced cell apoptosis by targeting the BH4 domain to induce the exposure of the Bcl-2 BH3 domain and result in the conformational changes of Bcl-2 but not regulating the expression of Mcl-1 in HUVECs. In addition, we found that treatment with the same concentration of CYD0281 and BDA-366 resulted in greater exposure of the Bcl-2 BH3 domain in the CYD0281 group than that in the BDA366-treated group. Interestingly, BDA-366 was found to show a more significant inhibitory effect on angiogenesis than CYD0281, indicating that such effects might be dependent on both switching Bcl-2 conformation and other relevant mechanisms. The effects of BDA-366 on Bcl-2 in vascular endothelial cells have not heretofore been investigated. BDA-366 negatively regulates the activity but not the expression of Bcl-2 in H460 non-small cell lung cancer cells [36]. In this study, BDA-366, but not CYD0281, also significantly inhibits the expression of Bcl-2 in vascular endothelial cells. In addition, the expression of Bcl-2 can be inhibited by BDA-366 in HT Bcl2 and G326 cells but not other chronic lymphocytic leukemia cells [66]. All the findings demonstrated that the regulatory mechanisms of BDA-366 on Bcl-2 may be cell-type dependent. However, the exact mechanisms of BDA-366 on inhibition of Bcl-2 expression in different cells remain to be further elucidated. In addition, the absorption efficiency of BDA-366 and CYD0281 by cells can also affect the intracellular drug concentration, thereby affecting its inhibitory effect on Bcl-2. Therefore, the potential mechanism that affects the inhibitory efficiency of CYD0281 on Bcl-2 in cells needs to be further elucidated through experiments. Our findings support that CYD0281 is a new antagonist that can be used to study the function of the Bcl-2 BH4 domain in vascular endothelial cells.

## Conclusion

We have discovered CYD0281, a new potent Bcl-2 BH4 inhibitor, which exhibits significant anti-angiogenic effects in vitro and in vivo. In addition, CYD0281 further exhibits an anti-cancer profile in the breast cancer experimental tumor model employed in the chick embryo CAM and 4T1 tumor mouse model. Moreover, CYD0281 induces the exposure of the BH3 domain and results in conformational changes of Bcl-2, thereby making Bcl-2 from an anti-apoptotic molecule to a pro-apoptotic killer that inhibits angiogenesis. The collective findings on this novel Bcl-2 BH4 antagonist suggest that CYD0281 may act as a powerful pharmacological tool for further elucidating the functions of Bcl-2 and its BH domains, and also serve as a potential therapeutic drug candidate for targeting neovascularization in breast cancer.

## Supplementary Information

**Additional file 1: Supplementary Figure 1.** Inhibition of the Bcl-2 BH4 domain using BDA-366 and CYD0281 promotes cell apoptosis and suppresses cell migration in HUVECs. **Supplementary Figure 2.** BDA-366 and CYD0281 inhibit angiogenesis in chick embryo CAM and YSM models. **Supplementary Figure 3.** BDA-366 and CYD0281 suppress tumor growth and angiogenesis in the chick embryo CAM model. **Supplementary Figure 4.** CYD0281 promotes MDA-MB-231 cell apoptosis by the exposure of the Bcl-2 BH3 domain. **Supplementary Figure 5.** Expression of Bcl-2 in normal tissues and Evaluation of in vivo toxicity CYD0281.

**Aditional file 2. **Original gels for WB.

## Data Availability

The datasets generated and/or analyzed during the current study are available in figshare repository (https://figshare.com/s/ed54d529d3f6bbf9e3ab).

## Abbreviations

Bcl-2: B-cell lymphoma 2
BH domain: Bcl-2 homology domain
IP: Immunoprecipitation
IF: Immunofluorescence
WB: Western blotting
CAM: Chorioallantoic membrane
YSM: Yolk sac membrane
VEGF: Vascular endothelial growth factor
HUVEC: Human umbilical vein vascular endothelium cell
PI: Propidium iodide
