# Supplementary material for: CYD0281, a Bcl-2 BH4 domain antagonist, inhibits tumor angiogenesis and breast cancer tumor growth
Source: BMC Cancer. 2023 May 26;23:479. doi: 10.1186/s12885-023-10974-4 (PMC10224611; doi:10.1186/s12885-023-10974-4)
Supplement: Supplementary file 1 — Additional file 1: Supplementary Figure 1. Inhibition of the Bcl-2 BH4 domain using BDA-366 and CYD0281 promotes cell apoptosis and suppresses cell migration in HUVECs. Supplementary Figure 2. BDA-366 and CYD0281 inhibit angiogenesis in chick embryo CAM and YSM models. Supplementary Figure 3. BDA-366 and CYD0281 suppress tumor growth and angiogenesis in the chick embryo CAM model. Supplementary Figure 4. CYD0281 promotes MDA-MB-231 cell apoptosis by the exposure of the Bcl-2 BH3 domain. Supplementary Figure 5. Expression of Bcl-2 in normal tissues and Evaluation of in vivo toxicity CYD0281. [file 12885_2023_10974_MOESM1_ESM.docx]

**Supplemental data**

**
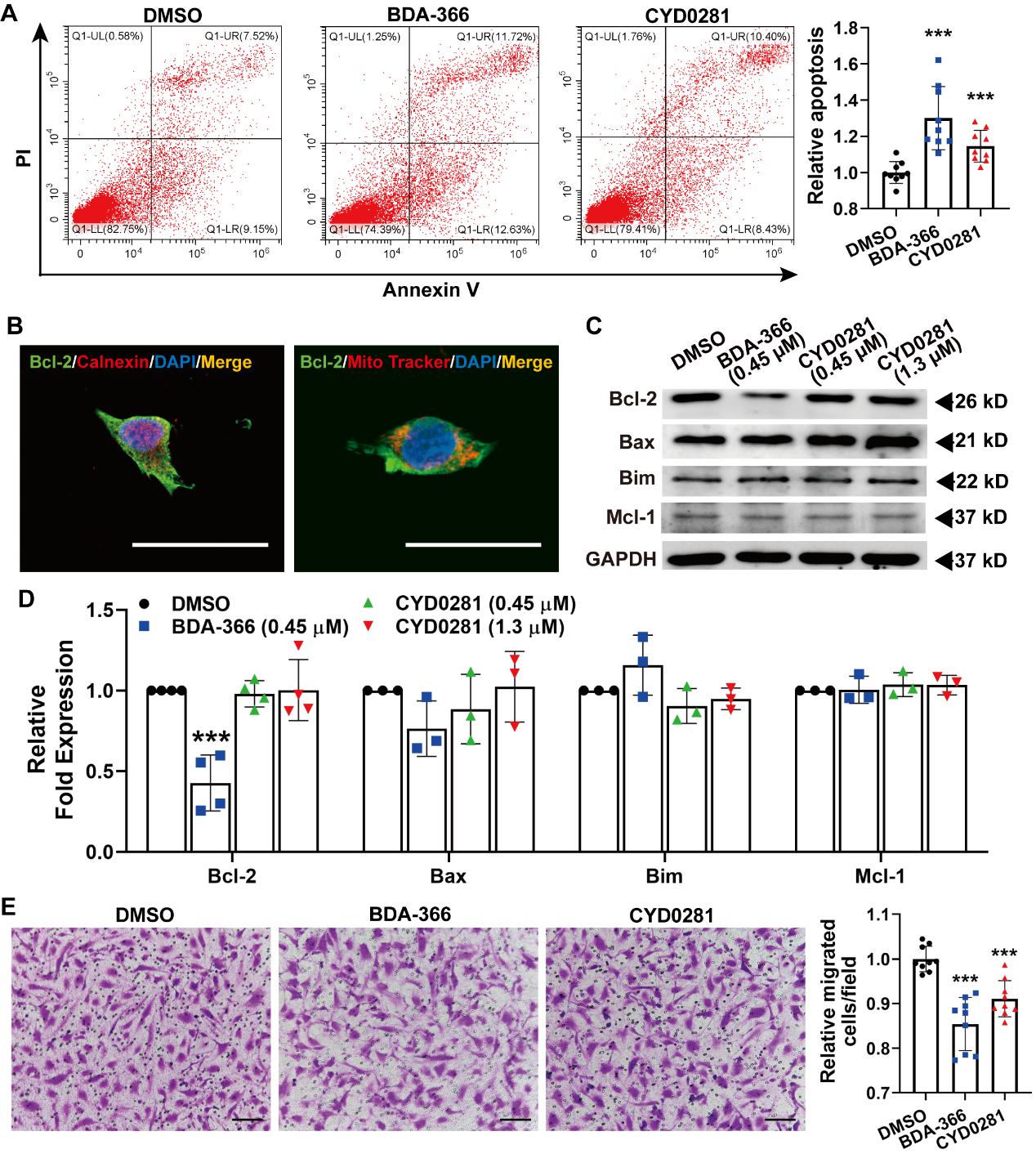
**

**Supplementary Figure 1.** **Inhibition of the Bcl-2 BH4 domain using BDA-366 and CYD0281 promotes cell apoptosis and suppresses cell migration in HUVECs.** HUVECs were treated with 0.1 μM of BDA-366 or CYD0281, a concentration in which cell viability was below 20%. (A) Representative flow cytometry diagram of cell apoptosis analysis of HUVECs treated with BDA-366 and CYD0281. Statistical analysis of relative cell apoptosis ratio. (B) Representative immunofluorescence staining images to label subcellular localization of Bcl-2 in the endoplasmic reticulum (ER), mitochondria, and cytosol. Calnexin and Mito-Tracker were used as the ER and mitochondrial markers, respectively (Scale Bar: 50 μm). (C and D) Bcl-2, Bax, Bim, and Mcl-1 protein expression in HUVECs treated with BDA-366 and CYD0281 at the IC_50_ concentration for 48 h. (D) Statistical analysis of western blotting. (E) Representative cell migration after treatment with DMSO, BDA-366, or CYD0281. Statistical analysis of the relative cell apoptosis ratio, the migrated cells were significantly inhibited by BDA-366 and CYD0281 compared with the effect of DMSO. (Scale Bar: 100 μm). The statistical analysis results are expressed as the mean ± SD. n=9. Significant effect: ****P*<0.001.

**
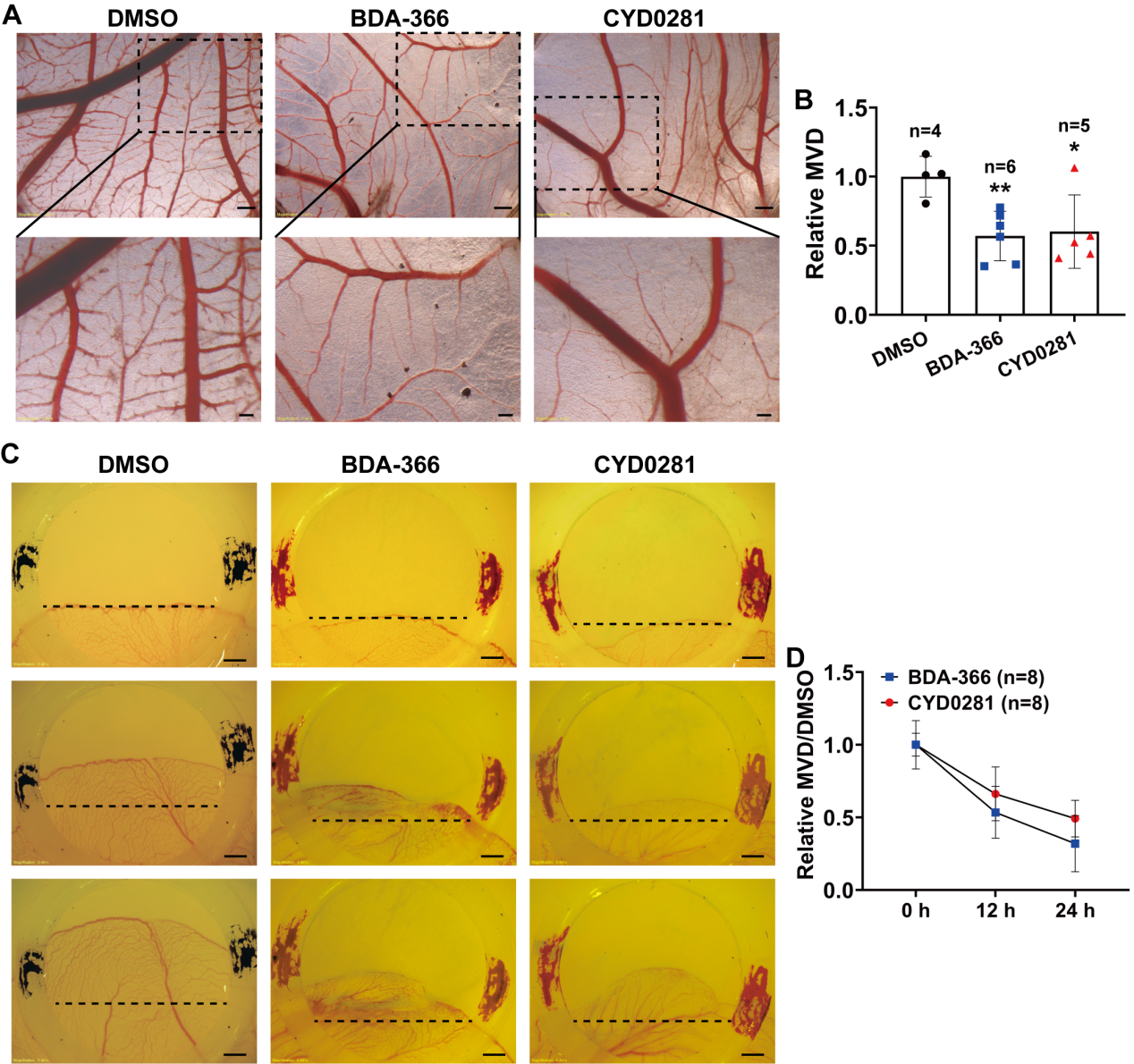
**

**Supplementary Figure 2. BDA-366 and CYD0281 inhibit angiogenesis in chick embryo CAM and YSM models.** Representative images of the growth of blood vessel branches and vascular network in DMSO-, BDA-366- and CYD0281 (1 μg, a concentration of 0.1% of xenograft tumor model treatment)-treated chick embryo CAM (A) and YSM (C) models. Statistical analysis of relative MVD in chick embryo CAM (B) and YSM (D) models. The results are expressed as the mean ± SD. Significant effect: **P* < 0.05, and ***P* < 0.01. Scale bars: 500 μm (upper panel of A), 200 μm (bottom panel of A), and 1 mm (C).


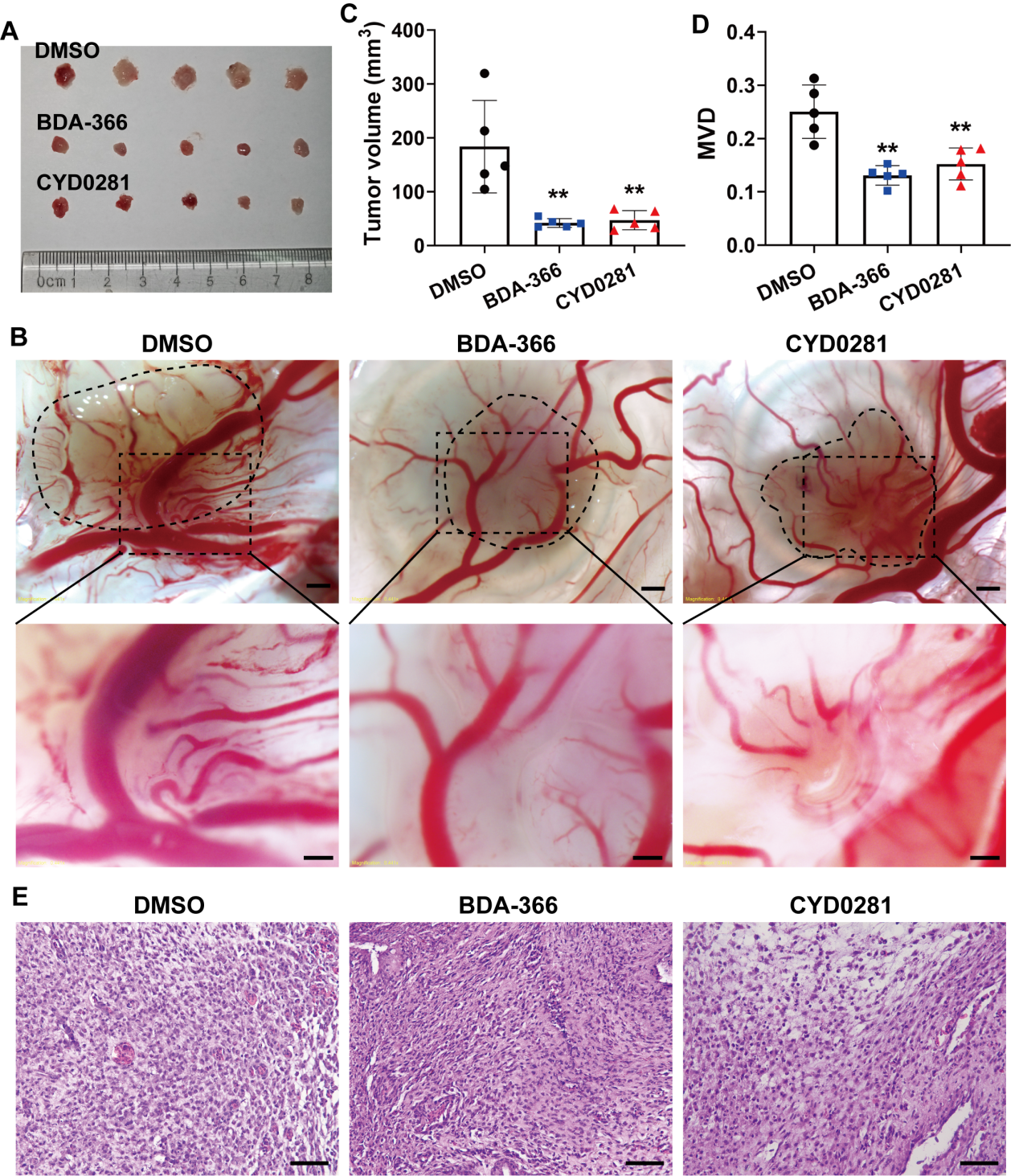


**Supplementary Figure 3.** **BDA-366 and CYD0281 suppress tumor growth and angiogenesis in the chick embryo CAM model.** (A) Representative images show the tumor following drug treatment. Tumors of DMSO-, BDA-366- and CYD0281-group were photographed macroscopically. (B) Representative images show the tumor of breast cancer xenografts in CAM treated with DMSO, BDA-366, or CYD0281 (1 μg/egg). Statistical analysis of tumor volume (C) and relative blood vessel density in the surface of tumor area (D). (E) Representative images of H&E staining of tumor tissues in chick embryo CAM. The results showed that treatment with BDA-366 and CYD0281 significantly reduced the number of blood vessels in tumor tissues compared with that in the DMSO group. The statistical analysis results are expressed as the mean ± SD. n=5. Significant effect: ** *P*<0.01. Scale bars: 1 mm (upper panel of B), 500 μm (bottom panel of B), and 50 μm (E).


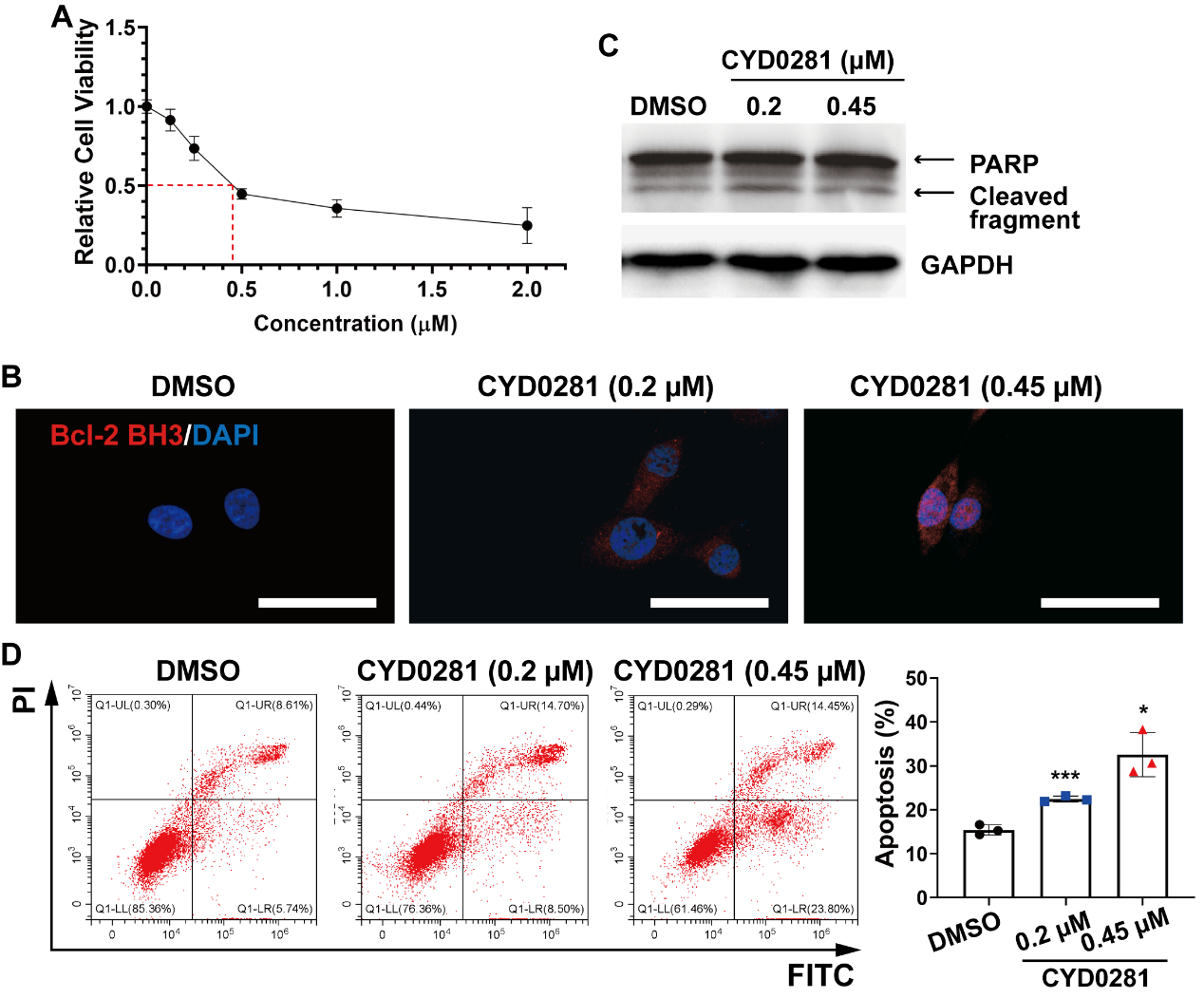


**Supplementary Figure 4. CYD0281 promotes** **MDA-MB-231 cell apoptosis by the exposure of the Bcl-2 BH3 domain.** (A) Cell viability of MDA-MB-231 cells at 48 h. The exposure of the BH3 domain of Bcl-2 detected by immunofluorescence (IF) staining using anti-Bcl-2/BH3 domain antibody (B) and PARP cleavage (C) were analyzed by western blotting assay and flow cytometry analysis of cell apoptosis (D) in MDA-MB-231 cell treated with CYD0281 (n=3). Significant effect compared to the DMSO group: **P*<0.05 and ****P*<0.001. Scale bars: 50 μm.


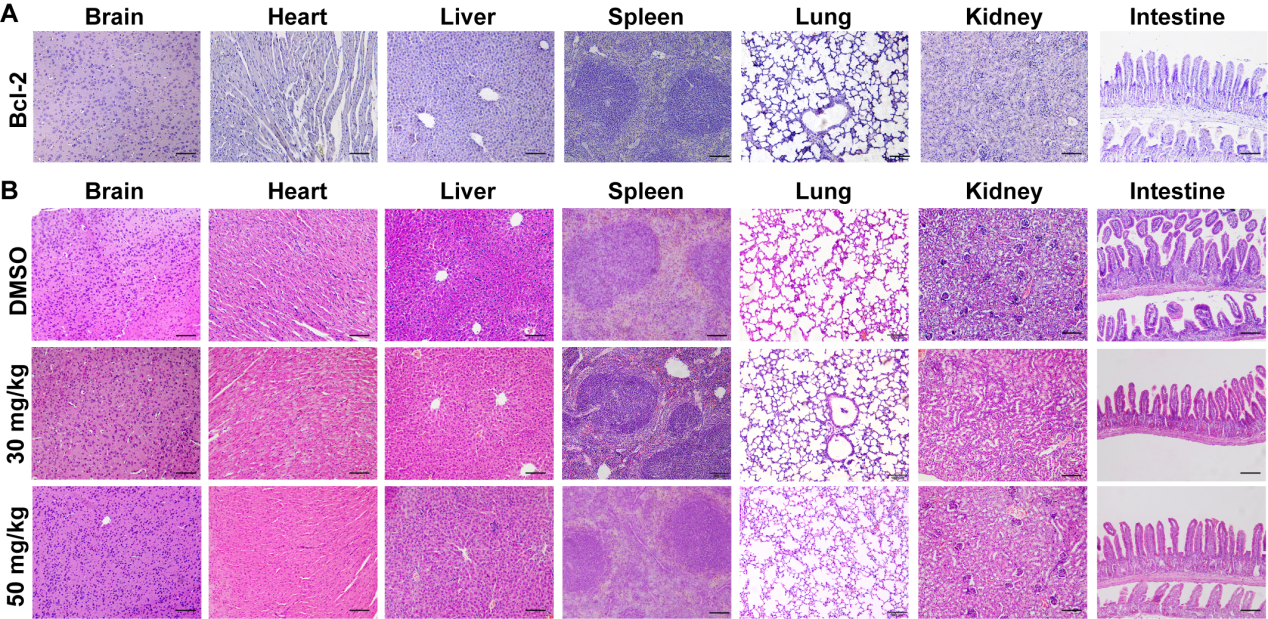


**Supplementary Figure 5. Expression of Bcl-2 in normal tissues and Evaluation of *in vivo* toxicity CYD0281.** (A) The expression of Bcl-2 protein in various organs of wild-type mice. No significant expression of Bcl-2 in normal tissues of wild-type mice. (B) H&E staining of histological sections from various organs of tumor model mice after treatment of CYD0281 using the dose of 30- and 50- mg/kg body weight. Scale bar: 100 μm.
