# Supplementary material for: CYD0281, a Bcl-2 BH4 domain antagonist, inhibits tumor angiogenesis and breast cancer tumor growth
Source: BMC Cancer. 2023 May 26;23:479. doi: 10.1186/s12885-023-10974-4 (PMC10224611; doi:10.1186/s12885-023-10974-4)

The original gels of Figure 1C. (A-C) The exposure of the BH3 domain of recombinant Bcl-2 protein was analyzed by immunoprecipitation using Bcl-2 BH3 specific antibody followed by Western blot analysis in three separate experiments.

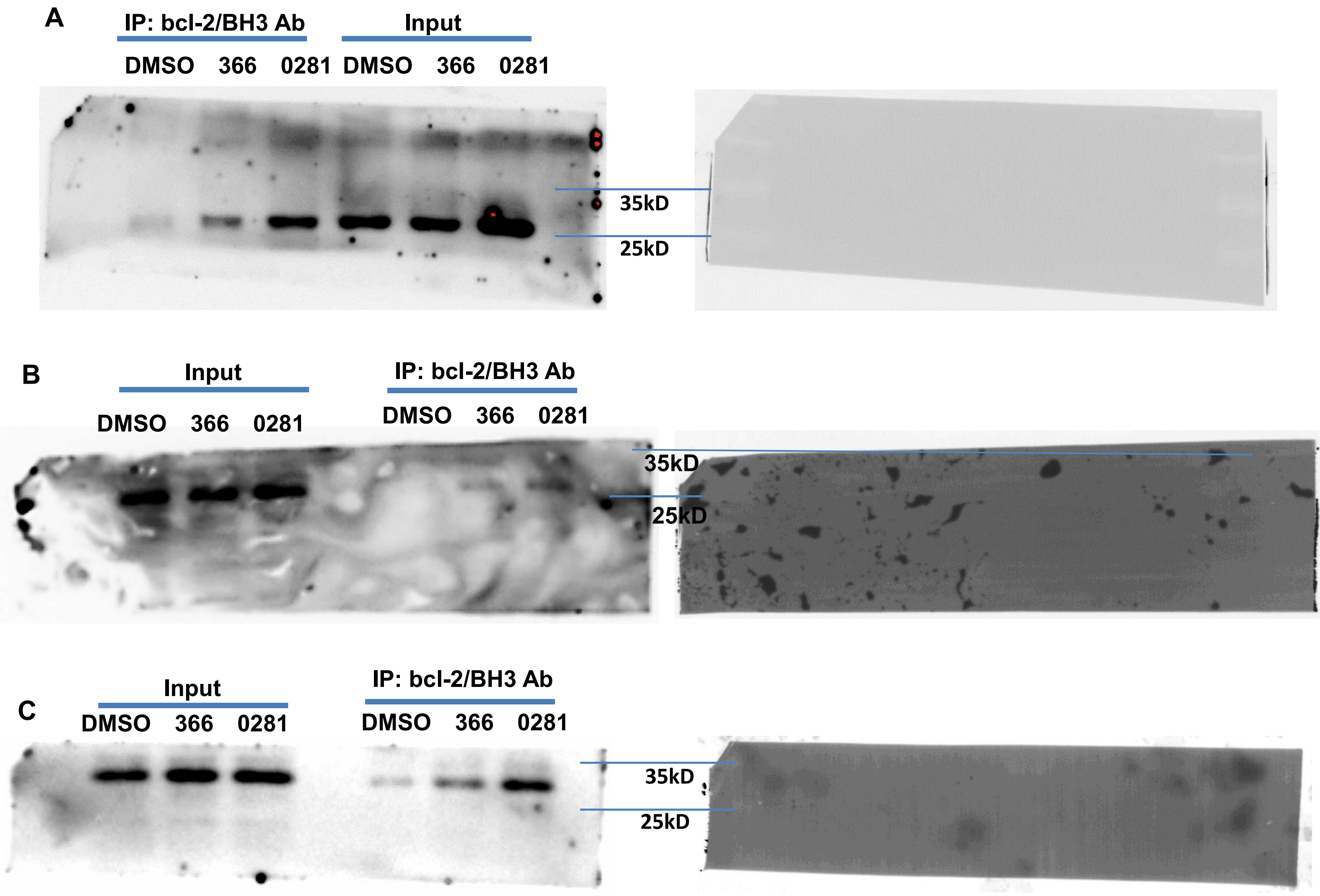

Supplement: Supplementary file 2 — Aditional file 2. Original gels for WB. [file 12885_2023_10974_MOESM2_ESM.zip › Figure 1C IP original gels.pdf]
