# Supplementary material for: CYD0281, a Bcl-2 BH4 domain antagonist, inhibits tumor angiogenesis and breast cancer tumor growth
Source: BMC Cancer. 2023 May 26;23:479. doi: 10.1186/s12885-023-10974-4 (PMC10224611; doi:10.1186/s12885-023-10974-4)

The original gels of Figure 2E. The Cyt c release was analyzed by western blotting assay. (A) The original gels of Cyt c release in mitochondria and cytosol in CYD0281-treated HUVECs. (B) The original gels of GAPDH expression in extracted proteins of mitochondria and cytosol.

A

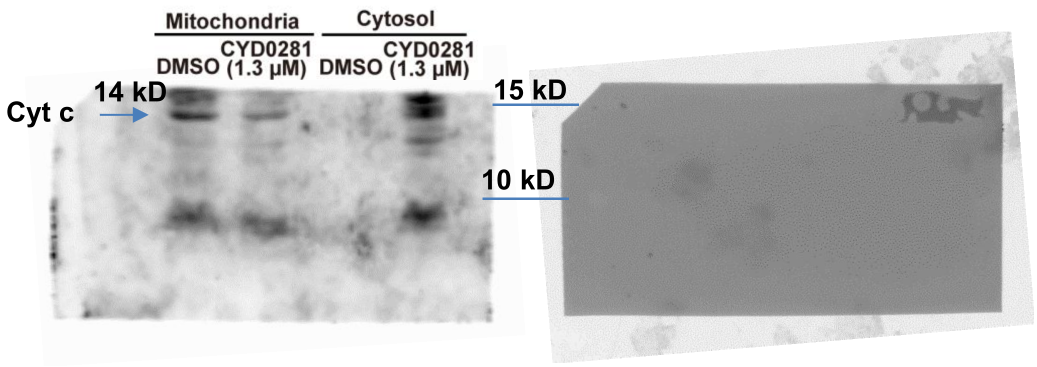

B

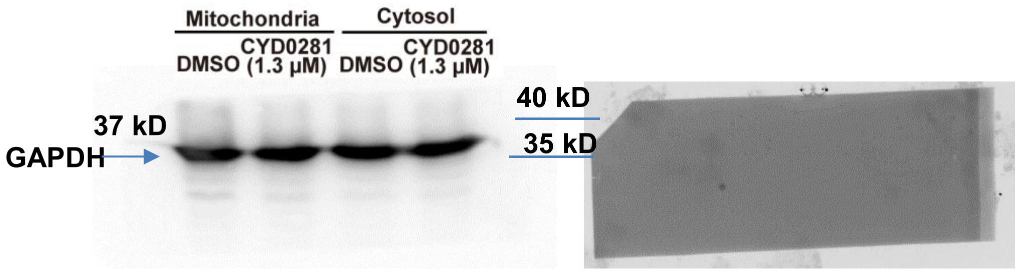

Supplement: Supplementary file 2 — Aditional file 2. Original gels for WB. [file 12885_2023_10974_MOESM2_ESM.zip › revised Figure 2E original gels.pdf]
