# Supplementary material for: CYD0281, a Bcl-2 BH4 domain antagonist, inhibits tumor angiogenesis and breast cancer tumor growth
Source: BMC Cancer. 2023 May 26;23:479. doi: 10.1186/s12885-023-10974-4 (PMC10224611; doi:10.1186/s12885-023-10974-4)

The original gels of Figure 2G. A co-IP using Bcl-2 antibody were performed following treatment of CYD0281 in HUVECs, and the interaction between Bcl-2 and Bax or Bim was analyzed by western blotting assay. The original gels of western blotting assay of Bcl-2 (A), Bax (B) and Bim (C).

A

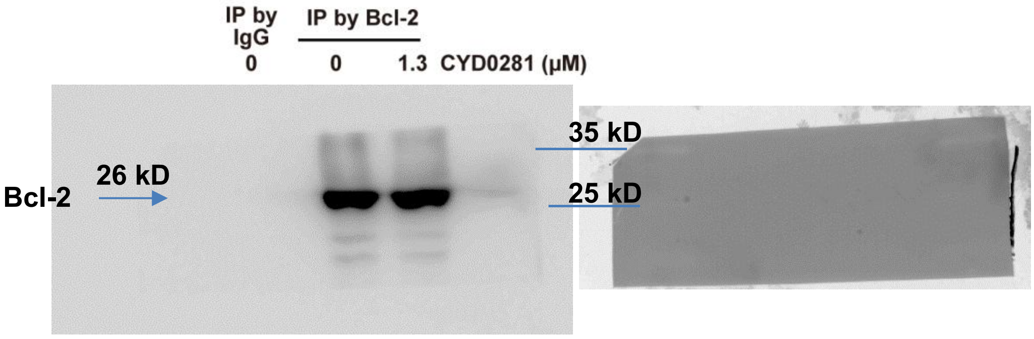

B

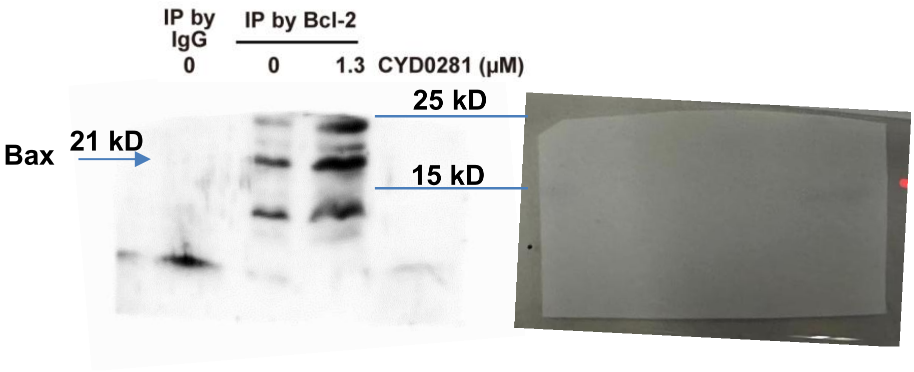

C

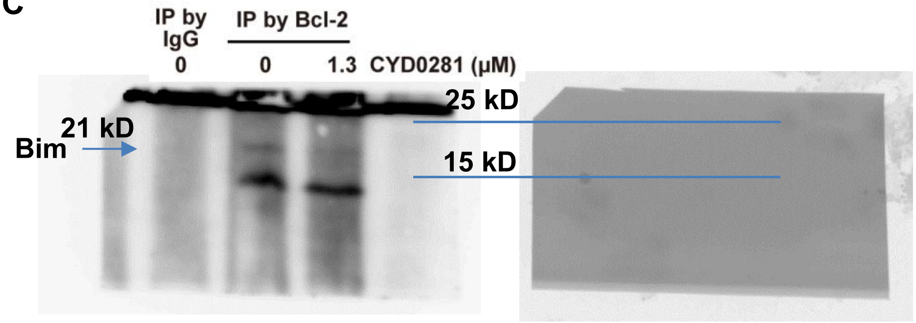

Supplement: Supplementary file 2 — Aditional file 2. Original gels for WB. [file 12885_2023_10974_MOESM2_ESM.zip › revised Figure 2G original gels.pdf]
