# Supplementary material for: CYD0281, a Bcl-2 BH4 domain antagonist, inhibits tumor angiogenesis and breast cancer tumor growth
Source: BMC Cancer. 2023 May 26;23:479. doi: 10.1186/s12885-023-10974-4 (PMC10224611; doi:10.1186/s12885-023-10974-4)

The original gels of Figure 2H. The expression of Bcl-2 in Bcl-2 siRNAs transfected HUVECs analyzed by western blotting assay. The original gels of Bcl-2 (A) and GAPDH expression (B) in HUVECs transfected with Bcl-2 siRNAs.

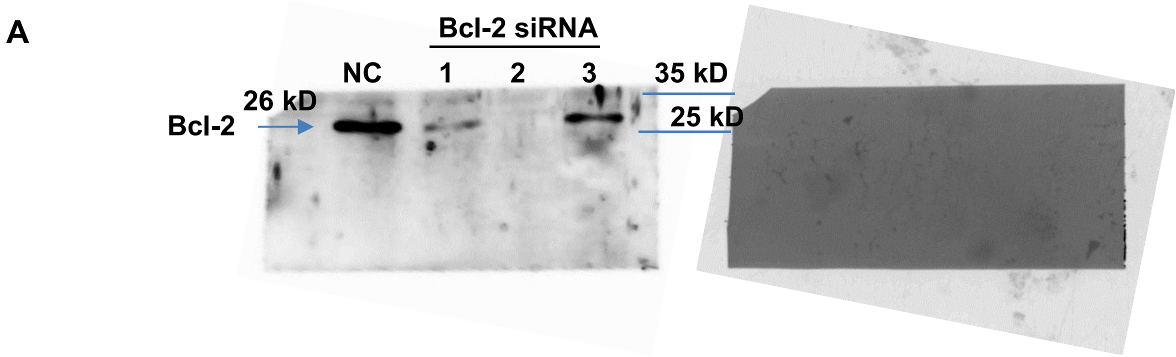

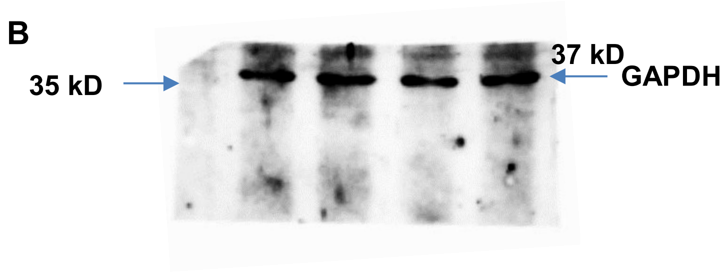

Supplement: Supplementary file 2 — Aditional file 2. Original gels for WB. [file 12885_2023_10974_MOESM2_ESM.zip › revised Figure 2H original gels.pdf]
