# Supplementary material for: CYD0281, a Bcl-2 BH4 domain antagonist, inhibits tumor angiogenesis and breast cancer tumor growth
Source: BMC Cancer. 2023 May 26;23:479. doi: 10.1186/s12885-023-10974-4 (PMC10224611; doi:10.1186/s12885-023-10974-4)
Supplement: Supplementary file 2 — Aditional file 2. Original gels for WB. [file 12885_2023_10974_MOESM2_ESM.zip › revised supplementary Figure 1C original gels.pdf]

**The original gels of Supplementary Figure 1C.** The original gels of Mcl-1 (A-C), Bcl-2 (D-F), Bax (G-I), and Bim (J-L) protein expression in HUVECs treated with BDA-366 and CYD0281 at the IC$_{50}$ concentration for 48 h in three separate experiments.

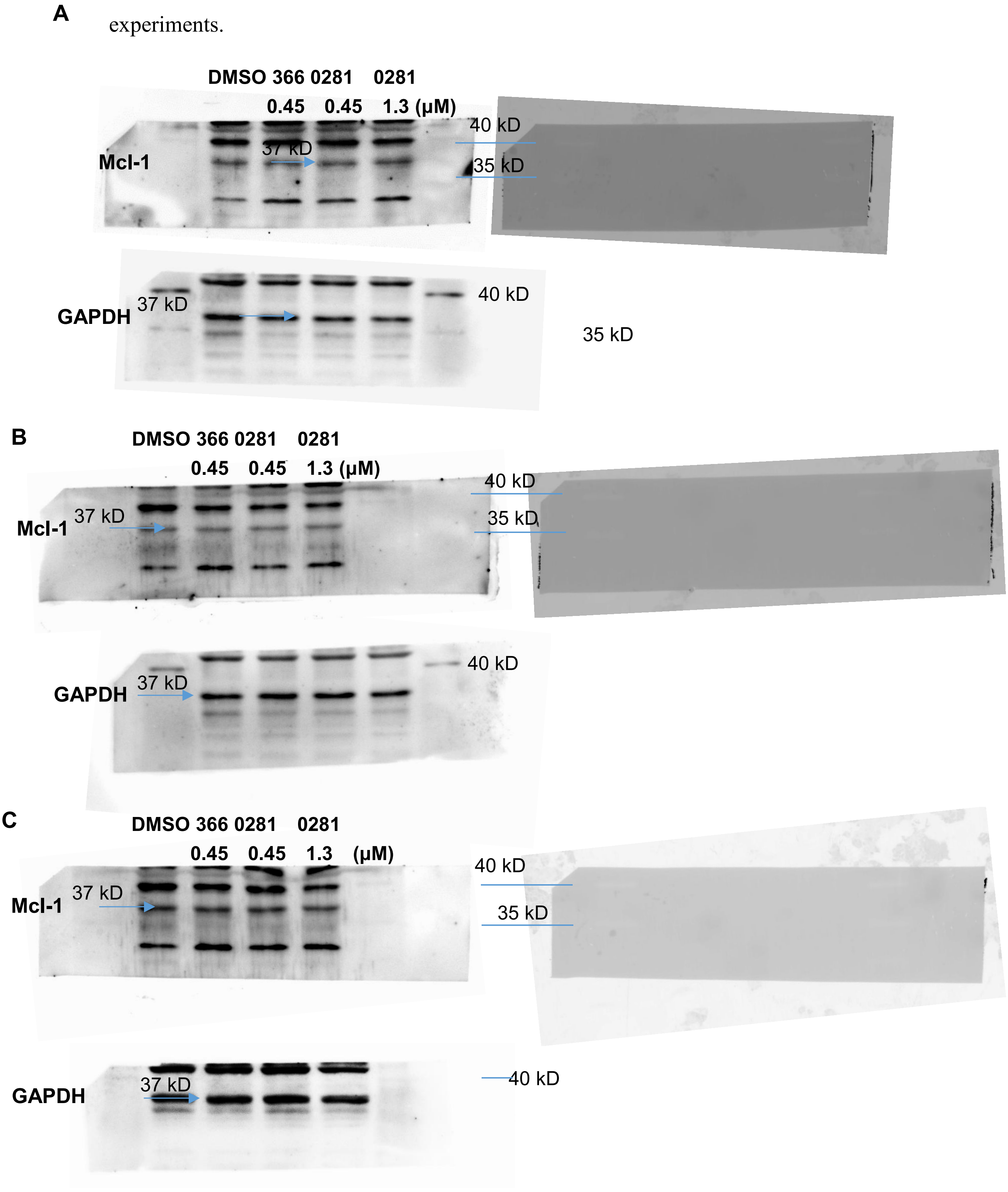

D

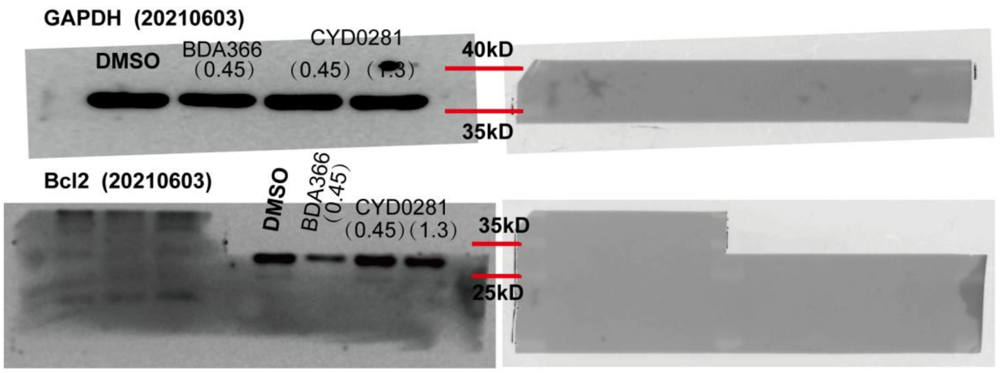
GAPDH (20210603)
DMSO
BDA366 (0.45)
CYD0281 (0.45) (1.3)
40kD
35kD
Bcl2 (20210603)
DMSO
BDA366 (0.45)
CYD0281 (0.45) (1.3)
35kD
25kD

E

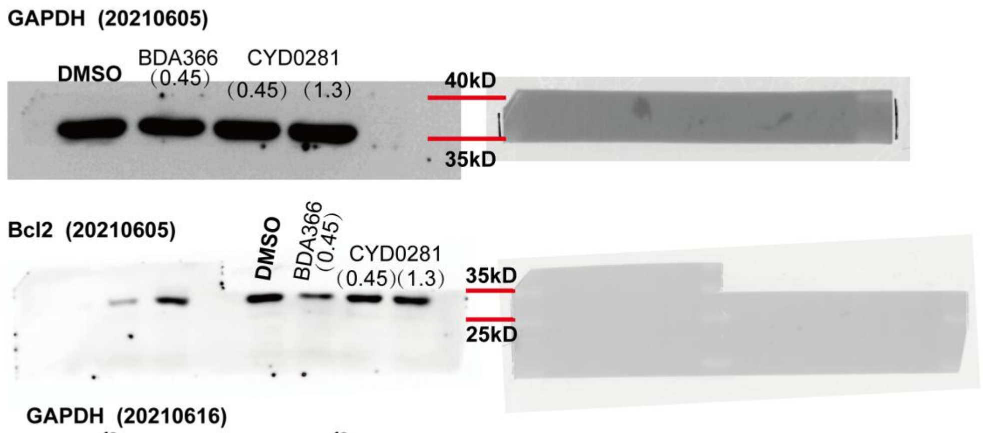
GAPDH (20210605)
DMSO
BDA366 (0.45)
CYD0281 (0.45) (1.3)
40kD
35kD
Bcl2 (20210605)
DMSO
BDA366 (0.45)
CYD0281 (0.45) (1.3)
35kD
25kD

F

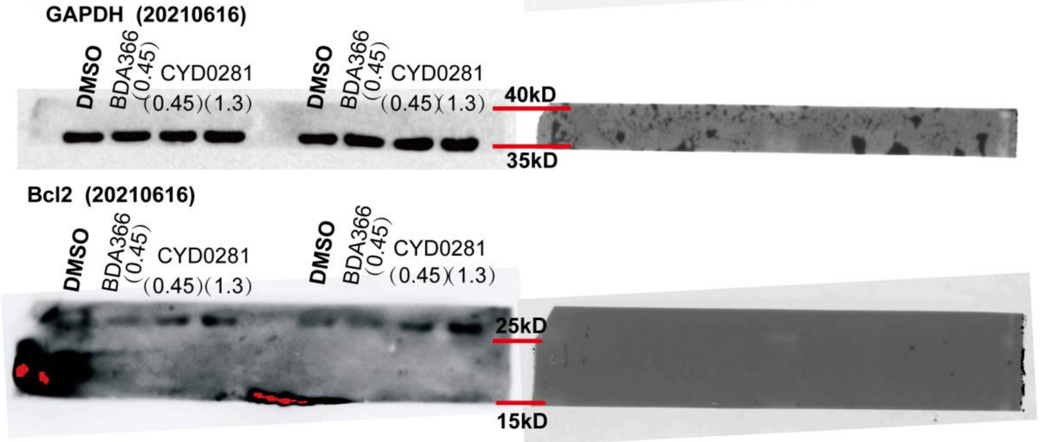
GAPDH (20210616)
DMSO
BDA366 (0.45)
CYD0281 (0.45) (1.3)
DMSO
BDA366 (0.45)
CYD0281 (0.45) (1.3)
40kD
35kD
Bcl2 (20210616)
DMSO
BDA366 (0.45)
CYD0281 (0.45) (1.3)
DMSO
BDA366 (0.45)
CYD0281 (0.45) (1.3)
25kD
15kD

G

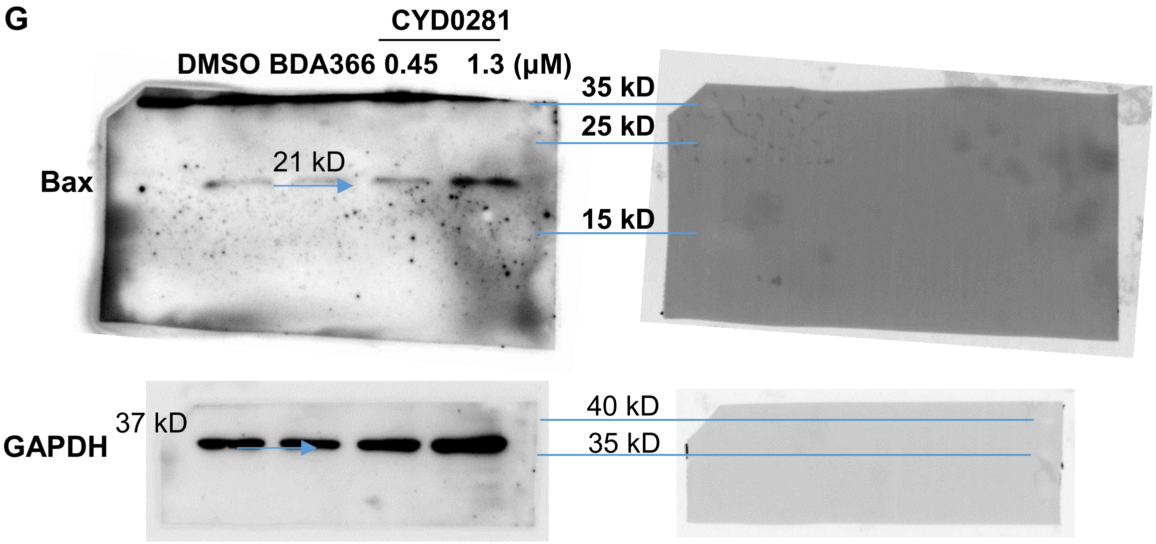
CYD0281
DMSO BDA366 0.45 1.3 (μM)
35 kD
25 kD
15 kD
Bax
21 kD
GAPDH
37 kD
40 kD
35 kD

H

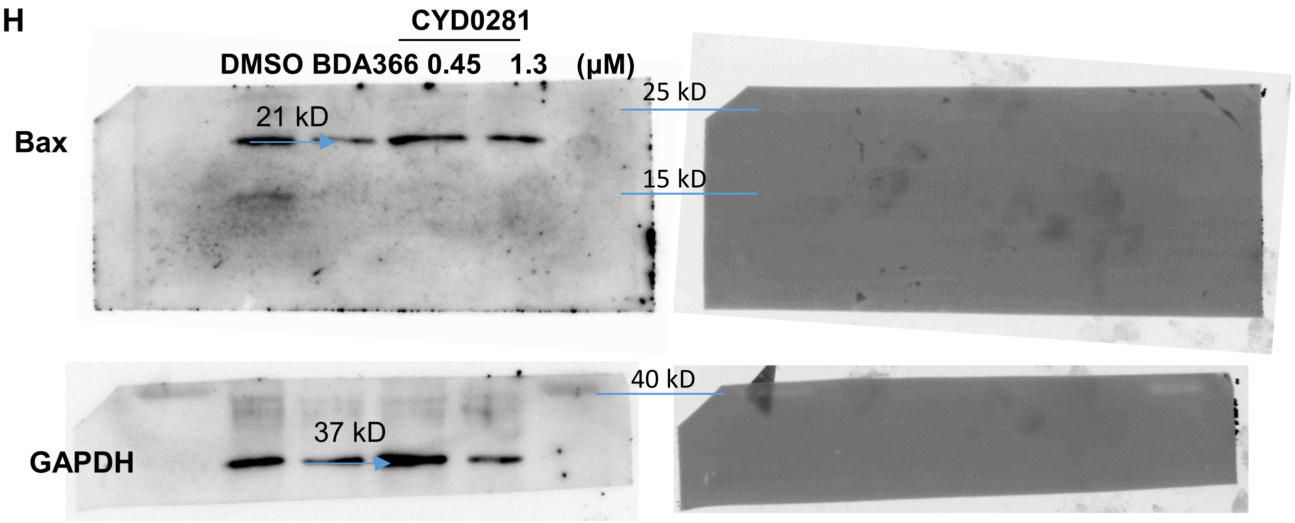
CYD0281
DMSO BDA366 0.45 1.3 (μM)
25 kD
15 kD
Bax
21 kD
GAPDH
37 kD
40 kD

I

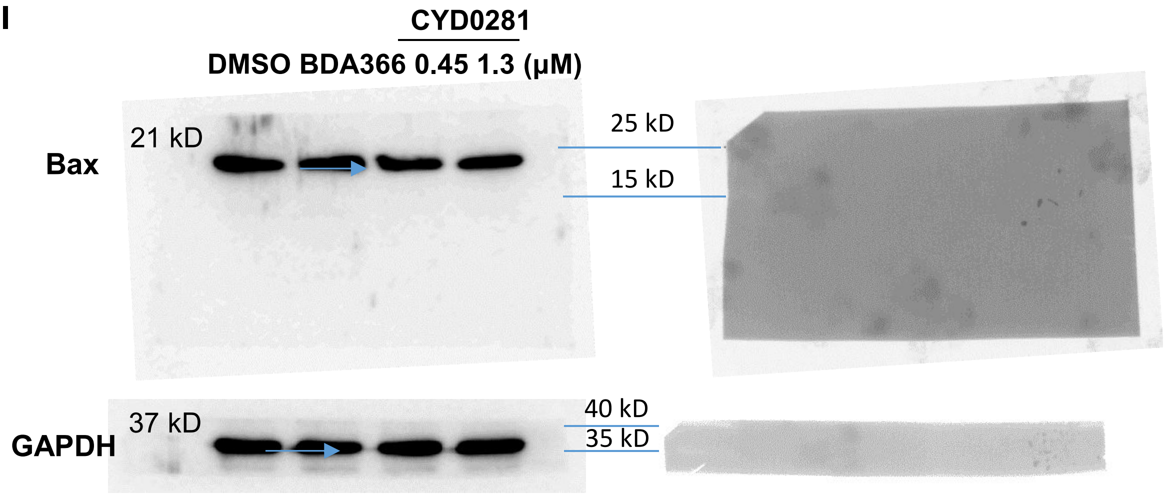
CYD0281
DMSO BDA366 0.45 1.3 (μM)
Bax
21 kD
25 kD
15 kD
GAPDH
37 kD
40 kD
35 kD

J

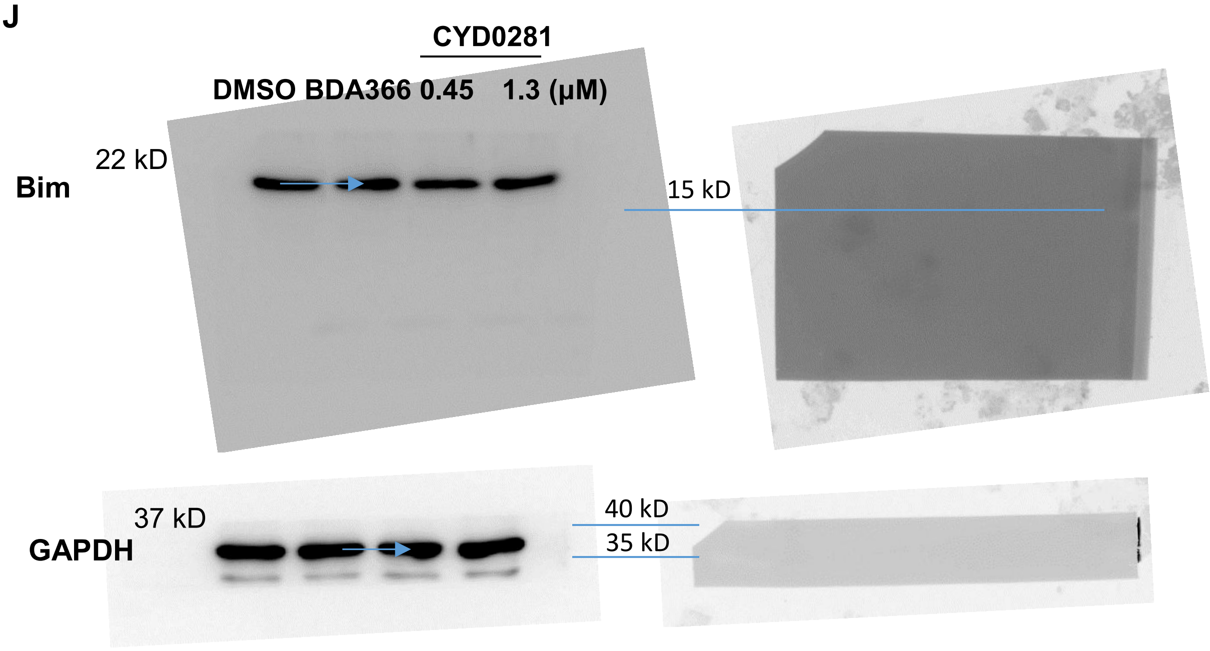
CYD0281
DMSO BDA366 0.45 1.3 (μM)
22 kD
Bim
15 kD
37 kD
GAPDH
40 kD
35 kD

K

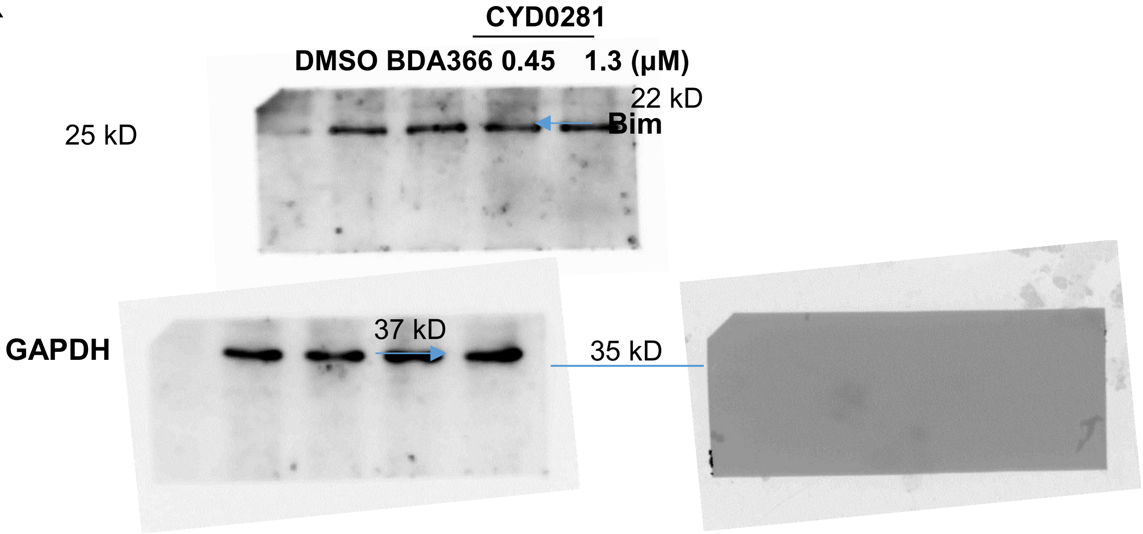
CYD0281
DMSO BDA366 0.45 1.3 (μM)
25 kD
22 kD
Bim
GAPDH
37 kD
35 kD

L

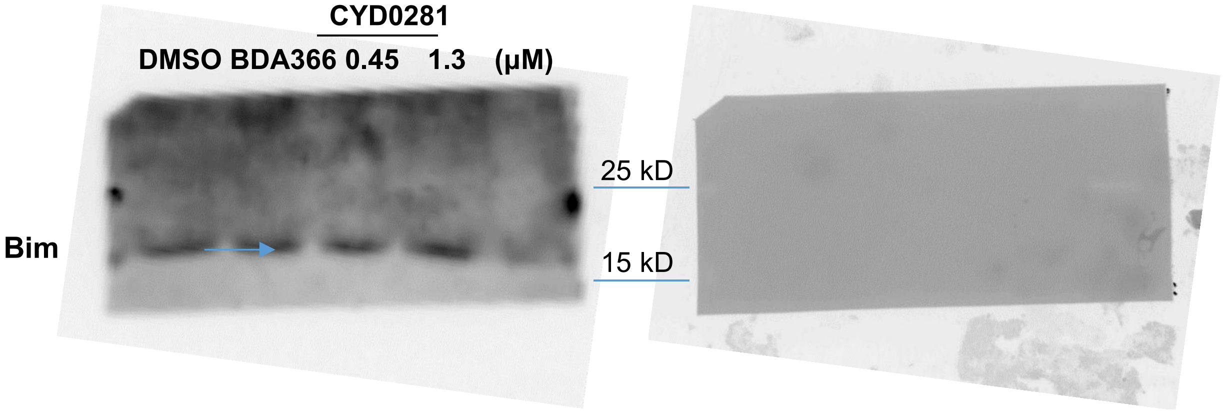
CYD0281
DMSO BDA366 0.45 1.3 (μM)
Bim
25 kD
15 kD

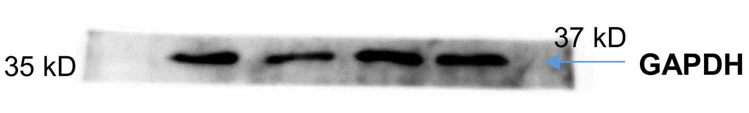
35 kD
37 kD
GAPDH
